# Progress and Promise of Nitric Oxide‐Releasing Platforms

**DOI:** 10.1002/advs.201701043

**Published:** 2018-04-23

**Authors:** Tao Yang, Alexander N. Zelikin, Rona Chandrawati

**Affiliations:** ^1^ School of Chemical Engineering The University of New South Wales (UNSW Sydney) Sydney NSW 2052 Australia; ^2^ School of Chemical and Biomolecular Engineering The University of Sydney Sydney NSW 2006 Australia; ^3^ Department of Chemistry and iNANO Interdisciplinary Nanoscience Center Aarhus University Aarhus C 8000 Denmark

**Keywords:** biomaterials, enzyme mimics, enzyme prodrug therapy, nitric oxide, nitric oxide donors

## Abstract

Nitric oxide (NO) is a highly potent radical with a wide spectrum of physiological activities. Depending on the concentration, it can enhance endothelial cell proliferation in a growth factor‐free medium, mediate angiogenesis, accelerate wound healing, but may also lead to tumor progression or induce inflammation. Due to its multifaceted role, NO must be administered at a right dose and at the specific site. Many efforts have focused on developing NO‐releasing biomaterials; however, NO short half‐life in human tissues only allows this molecule to diffuse over short distances, and significant challenges remain before the full potential of NO can be realized. Here, an overview of platforms that are engineered to release NO via catalytic or noncatalytic approaches is presented, with a specific emphasis on progress reported in the past five years. A number of NO donors, natural enzymes, and enzyme mimics are highlighted, and recent promising developments of NO‐releasing scaffolds, particles, and films are presented. In particular, key parameters of NO delivery are discussed: 1) NO payload, 2) maximum NO flux, 3) NO release half‐life, 4) time required to reach maximum flux, and 5) duration of NO release. Advantages and drawbacks are reviewed, and possible further developments are suggested.

## 1 Introduction

Nitric oxide (NO) is a highly potent two‐atom radical with a wide spectrum of physiological activities. In 1992, NO was crowned the “Molecule of the Year”^1^ and in 1998 Robert Furchgott, Louis Ignarro, and Ferid Murad shared the Nobel Prize in Physiology or Medicine for their significant discoveries on NO as a signaling molecule in the cardiovascular system. NO has been the focus of immense scientific and medical research, and is recognized as a versatile player in nearly every physiological system: cardiovascular,^2^ immune,^3^ central nervous system,^4^ and outflow physiology.^5^ In the body, NO at varied concentrations (nm–µm) is produced intracellularly by the enzymatic action of NO synthase (NOS) from amino acid l‐arginine. Several isoforms of NOS exist, including endothelial NOS (eNOS), neuronal NOS (nNOS), and inducible NOS (iNOS). Healthy endothelial cells produce NO at a flux of 0.05–0.40 nmol min^−1^ cm^−2^ 6, 7 (NO flux denotes the amount of NO flows in certain areas at a defined timeframe). NO can also be generated through non‐NOS pathways, i.e., via conversion of nitrite ions to NO. In the cardiovascular system, NO signals the surrounding smooth muscle to relax, leading to vasodilation (widening of blood vessels) and increasing blood flow.^2^ NO influences angiogenesis and vascular remodeling,^8^ transmits neural messages,^9^ and aids in the killing of various pathogens, i.e., bacteria and parasites.^10^ This radical affects an early step in the replication cycle of influenza viruses and NO has been shown to severely impair the replication of influenza A and B viruses.^11^ In the outflow physiology, NO has been reported to contribute to the physiological regulation of aqueous humor outflow and to lower intraocular pressure in various animal models and human patients.^5^, ^12^, ^13^, ^14^, ^15^

Due to the significance and therapeutic potential of NO, many efforts have focused on developing feasible means for effective NO delivery. However, the quests have been met by several challenges. To date, there are only a few US Food and Drug Administration (FDA) approved products on the market for NO delivery, for example, nitroglycerine for the treatment of acute angina,^16^ Nitropress (nitroprusside) for the treatment of congestive heart failure and life‐threatening high blood pressure,^17^ inhaled NO for pulmonary treatments,^18^ and VYZULTA (latanoprostene bunod ophthalmic solution) to lower intraocular eye pressure in glaucoma patients.^19^ NO is a simple molecule but with complex actions. NO activity is tissue‐specific and it can exert protective or deleterious effects depending on its concentration^20^, ^21^ (**Table** **1**); lower NO concentrations (nm) generally promote cell survival and proliferation, while higher concentrations (µm) lead to apoptosis and cell cycle arrest. NO passively diffuses in tissues across distances of ≈100 µm to red blood cells, where it reacts rapidly with oxygenated hemoglobin and myoglobin to produce nitrate.^22^, ^23^ This reaction results in the rapid scavenging of NO and serves as a mechanism to maintain NO homeostasis in the vascular compartment. In the context of drug delivery, as NO disappears within seconds in the blood, its short biological half‐life severely limits its efficacy from reaching a target site. NO can also react with superoxide radicals (O_2_^−^) to form a powerful oxidant peroxynitrite (ONOO^−^), which induces DNA damage and lipid peroxidation.^24^, ^25^ The inactivation of NO by O_2_^−^ creates intrinsic NO deficiency, which can lead to pathological conditions such as atherosclerosis,^26^ blood flow disturbances in the central nervous system,^27^ inflammation,^28^ poor wound healing,^8^ and tumor progression.^29^ Due to its multifaceted role, some effects of NO may be counterproductive and oppose the desired therapeutic outcome, and more does not mean better. Therefore, NO must be administered at the diseased site, in the right dose and at the right time.

**Table 1:** Effects of NO concentration on physiological activities^20^

NO concentration [nm]	Physiological results
<1–30	Promotes cell survival and proliferation
30–60	Protects cells from apoptosis
100	Protects tissue from injury
400	Mediates cell cycle arrest
>1000	Apoptosis and full cycle arrest, kills bacterial biofilms

**Figure 1:**
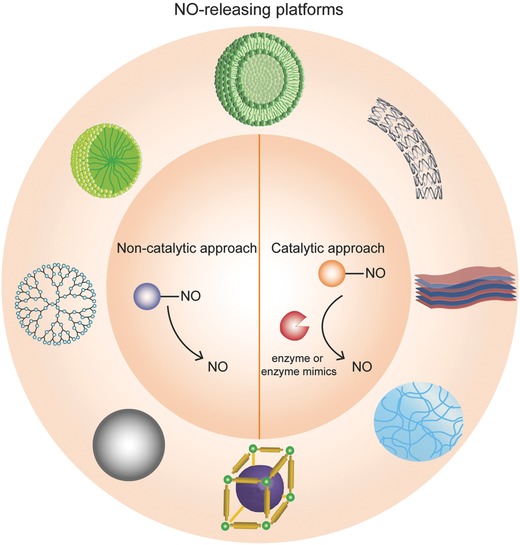
An overview of different platforms that are engineered to release NO (counterclockwise from center: liposomes, micelles, dendrimers, particles, metal–organic frameworks, hydrogels, freestanding films, implantable devices). NO can be delivered from encapsulated NO donors, which carry NO and stabilize the radical until release is required. NO can also be delivered via enzymatic conversion of custom‐made synthetic NO prodrugs or endogenous NO donors (using natural enzymes or nonproteinaceous enzyme mimics).

In this review, we discuss NO‐releasing platforms, with a specific emphasis on the combination of NO donors and bio(nano)materials. These platforms have emerged as a promising approach to overcome the challenges associated with biological administration of NO and to promote the spatiotemporal generation of physiologically relevant concentrations of NO in diverse biomedical applications. We highlight recent developments of materials (including scaffolds, particles, and films) for NO delivery via catalytic and noncatalytic approaches reported in the past five years (**Figure** **1**). Advantages and drawbacks of the NO‐releasing platforms are reviewed. In particular, five key parameters of NO delivery are discussed and compared: 1) NO payload, 2) maximum NO flux, 3) NO release half‐life (*t*_1/2_, time for materials to release half of its NO payload), 4) time required to reach maximum flux, and 5) duration of NO release. We begin with small molecule NO donors, liposomes, micelles, dendrimers, particles, and finally implantable biomaterials. Key parameters of NO delivery are summarized in **Table** **2**.

**Table 2:** NO release properties from a number of platforms at physiological conditions (37 °C, pH 7.4)

Materials	[NO]_total_ a) [µmol mg^−1^]	[NO]_max_ b)	*t*_1/2_ c) [min]	*t*_max_ d) [min]	*t*_d_ e) [h]	Ref.
Liposomes						
Liposome‐DPTA/NO	0.26 ± 0.05	29 ± 8 ppb mg^−1^	1224 ± 162	N/A	65.9 ± 1.8	37
Liposome‐PROLI/NO	5.10 ± 0.51f)	N/A	9.6 ± 3.0	N/A	2.8 ± 0.1	38
Liposome‐DEA/NO	9.16 ± 0.33f)	N/A	18.6 ± 1.2	N/A	4.6 ± 2.3	38
Liposome‐PAPA/NO	8.83 ± 0.64f)	N/A	156 ± 24	N/A	43.4 ± 3.9	38
Liposome‐SPER/NO	7.73 ± 0.71f)	N/A	2718 ± 276	N/A	168.2 ± 17.0	38
Dendrimers						
Alkyl‐modified G1 PAMAM dendrimers	1.06 ± 0.10	4930 ± 690 ppb mg^−1^	21 ± 5	1.9 ± 0.4	7 ± 2	50, 51
Alkyl‐modified G4 PAMAM dendrimers	0.91 ± 0.09	N/A	20 ± 3	1.5 ± 0.5	8 ± 4	51
QA/alkyl‐modified G1 PAMAM dendrimers	1.35 ± 0.30	8675 ± 8182 ppb mg^−1^	180 ± 30	1.0 ± 0.2	N/A	52
QA/alkyl‐modified G4 PAMAM dendrimers	1.48 ± 0.32	4550 ± 3097 ppb mg^−1^	228 ± 72	1.0 ± 0.2	N/A	52
PO/ED‐modified G1 PAMAM dendrimers	1.10 ± 0.18	5666 ± 562 ppb mg^−1^	52.2 ± 1.8	N/A	N/A	53
Silica particles						
Silica particles 30 nm diameter	0.88 ± 0.05	18.7 ± 2.2 ppm mg^−1^	27.4 ± 8.9	N/A	12.2 ± 3.0	59
Silica particles 150 nm diameter	1.30 ± 0.11	22.6 ± 4.4 ppm mg^−1^	40.7 ± 11.0	N/A	16.7 ± 1.4	59
Silica particles 450 nm diameter	0.82 ± 0.08	6.6 ± 1.8 ppm mg^−1^	88.2 ± 10.5	N/A	14.0 ± 0.3	59
Silica particles 1100 nm diameter	1.41 ± 0.19	32.8 ± 9.8 ppm mg^−1^	25.6 ± 5.0	N/A	11.1 ± 0.7	59
Silica particles with an aspect ratio of 1	0.76 ± 0.12	5400 ± 1100 ppb mg^−1^	46.2 ± 6.0	N/A	N/A	61
Silica particles with an aspect ratio of 4	0.69 ± 0.09	5000 ± 800 ppb mg^−1^	42.0 ± 5.4	N/A	N/A	61
Silica particles with an aspect ratio of 8	0.77 ± 0.13	5380 ± 700 ppb mg^−1^	45.6 ± 6.0	N/A	N/A	61
Polymer particles						
PLGA particle‐SNAP	0.3–0.6	N/A	N/A	N/A	240–336	72
Ester‐capped PLGA particle‐SNAP	0.3–0.6	N/A	N/A	N/A	720	72
Metal–organic frameworks						
Fe_2_(NO)_2_(dobdc)	4.0	N/A	N/A	N/A	240g)	79
Zn(2nIm)_2_	3.4	N/A	N/A	N/A	3h)	80
Zn(mnIm)_2_	2.9	N/A	N/A	N/A	3h)	80
Implantable devices						
CarboSil intravascular catheter‐SNAP	N/A	0.4 nmol cm^−2^ min^−1^	N/A	N/A	336	87
Dual‐lumen intravascular catheter‐SNAP	N/A	0.4 nmol cm^−2^ min^−1^	N/A	N/A	336	89
Silicone Foley catheter‐SNAP	N/A	0.4 nmol cm^−2^ min^−1^	N/A	N/A	720	90
Central venous catheter‐DBHD/NONO	N/A	0.4 nmol cm^−2^ min^−1^	N/A	N/A	336	88

^a)^ NO payload

^b)^ Maximum NO flux

^c)^ NO release half‐life

^d)^ Time required to reach maximum flux

^e)^ Duration of NO release

^f)^ Units reported in µmol mL^−1^

^g)^ NO release triggered by exposure to N_2_ at 11% relative humidity at 37 °C

^h)^ NO release triggered by light irradiation.

## 2 NO Delivery from Small Molecule NO Donors

NO donors are pharmacologically active substances that carry NO and stabilize the radical until release is required. There are several classes of NO donors, depending on their chemical reactivity or the mechanism of NO release from the carrier. The release of NO from donor molecules can be triggered by factors such as light, heat, pH changes, or enzyme activity. On a different route, NO can be released via chemical reactions of the donors with acids, alkalis, metals, or thiols.^30^ Readers are referred to excellent reviews that cover detailed aspects of NO donors.^31^, ^32^, ^33^ Due to their distinct advantages, a number of NO donors to note include nitrates, diazeniumdiolates (NONOates), and *S*‐nitrosothiols (RSNOs). NONOate is one of the most investigated NO donors due to its capability to release two moles of NO per mole of donor at physiological conditions and its pH‐dependent decomposition property. Generally, NONOates can be synthesized through reacting secondary amines with gaseous NO under high pressure (usually 5 atm). Structures with cationic primary amines can electrostatically stabilize the anionic diazeniumdiolate groups, leading to a range of NO release half‐life from 2 s to 20 h.^34^ A number of compounds in this group include: spermine NONOate, diethylamine NONOate, diethylenetriamine NONOate, dipropylenetriamine NONOate, and proline NONOate. Unlike NONOates, laboratory generation of RSNOs require reactions between thiols and nitrosating agents, such as alkyl nitrite, dinitrogen trioxide, and nitrous acid. NO can be exhausted from RSNOs by multiple triggers (i.e., heat, light, and copper ions). Two relatively stable compounds in this group and the most commonly used for in vivo preclinical studies include *S*‐nitroso‐*N*‐acetylpenicillamine (SNAP) and *S*‐nitrosoglutathione (GSNO).^31^

## 3 NO Delivery from Injectable Materials

To achieve functional NO‐releasing platforms, generally NO donors have to be available over the course of the envisaged therapeutic applications. NO donors must be stable enough at physiological conditions to reach the desired site, but are sufficiently labile under conditions unique to the target site. The clinical applications of low molecular weight NO donors have been restricted due to issues such as burst release and nontargeted delivery. Their encapsulation in carriers enabled controlled and sustained delivery of NO. A majority of studies on NO delivery from injectable materials to date have focused on their efficacy against bacteria and biofilms in vitro.

### 3.1 Liposomes

Liposomes are spherical vesicles made of amphiphilic lipids consisting of a hydrophobic shell and a hydrophilic core. Their sizes are typically in the range of 20 nm to 10 µm and they can be formed through techniques such as thin‐film hydration, solvent injection, reverse‐phase evaporation, sonication, membrane extrusion, and microfluidic technology.^35^, ^36^ The physical and chemical features of liposomes are highly dependent on the lipid composition. Suchyta and Schoenfisch encapsulated NO donors, spermine/NO (SPER/NO) or dipropylenetriamine/NO (DPTA/NO), within the aqueous core of dipalmitoylphosphatidylcholine (DPPC) liposomes using a reverse‐phase evaporation technique.^37^ SPER/NO and DPTA/NO belong to the NONOates, a class of NO donors that can spontaneously release NO at physiological conditions (37 °C, pH 7.4) due to donor breakdown by water (protonation). Encapsulation efficiency of NO donors within the liposomes was reported to be ≈35%. In contrast to free nonencapsulated SPER/NO, a ≈4.5‐fold increase in NO release half‐life, extending the *t*_1/2_ from 35 to 162 min, was achieved by the incorporation of SPER/NO in liposomes. The liposomal encapsulation of DPTA/NO, a more stable donor compared to SPER/NO, further slowed the NO release profile (*t*_1/2_ = 20 h). DPTA/NO‐based liposomes released NO continuously for ≈3 d. The authors also investigated *N*‐propyl‐1,3‐propanediamine/NO (PAPA/NO) in zwitterionic DPPC, dimyristoylphosphatidylcholine (DMPC), distearoylphosphatidylcholine (DSPC) liposomes; cationic dipalmitoyltrimethylammoniumpropane (DPTAP) liposomes; or anionic dipalmitoylphosphatidylglycerol (DPPG) liposomes.^38^ NO payload of 6–9 µmol mL^−1^ was obtained across the different liposome systems and duration of NO release could be tuned, from 18 h for DPTAP liposomes to 85 h for DSPC liposomes. Overall, this shows that: 1) the encapsulation of NO donors within the liposomes enables prolonged NO release due to the slow decomposition of the NO donors within the protective lipid bilayer and 2) NO release kinetics can be simply tuned by lipid composition and the type of NO donors encapsulated. NONOates decompose in a pH‐dependent manner and the release of NO from liposomes could be escalated under acidic conditions (pH 5.4), which can be applicable to the microenvironment of tumors.

**Figure 2:**
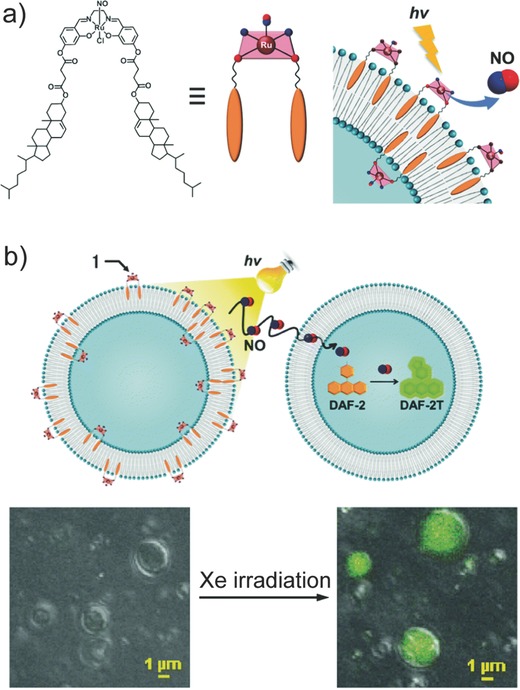
a) Chemical structure of a lipophilic photoresponsive NO donor, Ru nitrosyl complex [Ru(L)Cl(NO)] (L = *N*,*N*′‐ethylene‐bis(4‐cholesteryl‐hemisuccinate‐salicylideneamine)), and its incorporation into the bilayer of liposomes. b) Schematic illustration of NO transport from a [Ru(L)Cl(NO)]‐liposome to a 4,5‐diaminofluorescein (DAF‐2)‐liposome (top). NO release is triggered via Xe irradiation and the reaction of NO with DAF‐2 yields green fluorescent signal. Confocal laser scanning microscopy images of a mixture of [Ru(L)Cl(NO)]‐liposomes and DAF‐2‐liposomes before and after Xe irradiation (bottom). Reproduced with permission.^39^ Copyright 2015, The Royal Society of Chemistry.

Nakanishi et al. synthesized a lipophilic photoresponsive NO donor, Ru nitrosyl complex [Ru(L)Cl(NO)] (L = *N*,*N*′‐ethylene‐bis(4‐cholesteryl‐hemisuccinate‐salicylideneamine)), and attached the newly developed NO donor to the bilayer of 1,2‐ditetradecanoyl‐*sn*‐glycero‐3‐phospho‐(1′‐rac‐glycerol) liposomes, leading to the design of photoinduced NO‐releasing liposomes (**Figure** **2**a).^39^ Cholesterol anchoring into liposomes has been shown to be superior over a covalent linkage or electrostatic interaction,^40^, ^41^ and in this study cholesterol groups facilitated an effective linking affinity of the NO donors to the liposomes. Upon Xe irradiation, the liposomes released 2.3 × 10^−6^ m of NO, which is at a relevant concentration for anticancer activity. The authors further illustrated membrane transport of NO between coexisting liposomes. Liposomes encapsulating fluorescent reagent 4,5‐diaminofluorescein (DAF‐2) were mixed with [Ru(L)Cl(NO)] liposomes. Release of NO was triggered by Xe irradiation, and NO released crossed the lipid bilayer of DAF‐2 liposomes. Subsequently, DAF‐2 reacted with NO to yield green fluorescent DAF‐2T as shown by confocal microscopy imaging (Figure 2b).

### 3.2 Micelles

**Figure 3:**
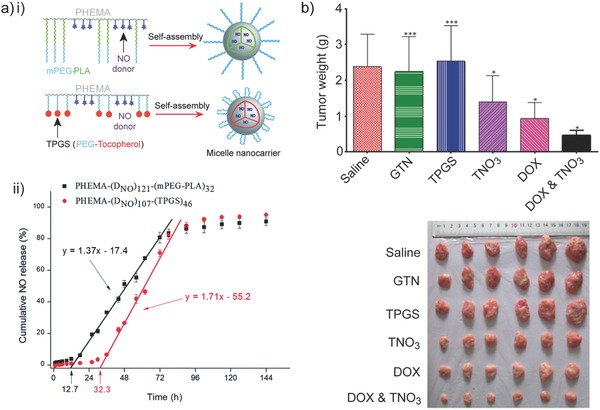
a) i) Schematic illustration of NO‐releasing poly(2‐hydroxyethyl methacrylate) (PHEMA) micelles with two different pendant moieties: methoxy poly(ethylene glycol) and poly(lactic acid) (m‐PEG‐PLA) or d‐α‐tocopheryl polyethylene glycol succinate (TPGS). (ii) Cumulative NO release from the two types of PHEMA micelles in PBS (pH 7.4) in the presence of 10 × 10^−3^ m glutathione. Reproduced with permission.^44^ Copyright 2015, The Royal Society of Chemistry. b) Tumor weight (top) and the corresponding photographs (bottom) of tumor‐bearing mice when treated with saline, nitroglycerine (GTN), TPGS, nitrate‐functionalized TPGS micelles (TNO_3_), anticancer drug doxorubicin (DOX), and the combination of DOX and TNO_3_. The codelivery of DOX and TNO_3_ leads to better antitumor efficacy. Reproduced with permission.^45^ Copyright 2014, American Chemical Society.

Polymeric micelles (10 to 200 nm) typically consist of amphiphilic polymers that self‐assemble to form a hydrophobic inner core stabilized by a hydrophilic outer shell.^42^, ^43^ Zhao and co‐workers fabricated NO‐containing poly(2‐hydroxyethyl methacrylate) (PHEMA) micelles with two different pendant moieties, methoxy poly(ethylene glycol) and poly(lactic acid) (m‐PEG‐PLA) or d‐α‐tocopheryl polyethylene glycol succinate (TPGS) (**Figure** **3**ai).^44^ NO donors (nitrates, which can decompose to liberate NO in the presence of reducing agents) were conjugated onto the hydroxyl groups of PHEMA and the graft copolymers assembled into NO‐loaded micelles with a size less than 100 nm spontaneously. A sigmodal NO release profile with two distinct phases (lag phase and plateau phase) was observed for both m‐PEG‐PLA‐modified and TPGS‐linked NO‐releasing micelles in phosphate buffered saline (PBS, pH 7.4) in the presence of 10 × 10^−3^ m glutathione (Figure 3aii). The onset of NO release from TPGS micelles was slower with a lag time of 32 h when compared to its mPEG‐PLA‐linked counterpart with a lag time of 13 h, illustrating that the choice of polymer building blocks can precisely tune NO release kinetics. TPGS micelles were designed for co‐delivery of NO and doxorubicin (DOX) to hepatocarcinoma HepG2 cancer cells, with synergistic effect between NO and DOX successfully induced higher cytotoxicity (≈6.25‐fold lower IC_50_).^45^ The antitumor activity was further explored on tumor‐bearing mice, and NO and DOX showed different tumor suppression effects compared to NO or DOX alone (Figure 3b).

### 3.3 Dendrimers

Dendrimers are 3D, well‐organized globular macromolecules with sizes ranging from 1 to 100 nm and consist of three distinct domains: 1) a central core, 2) a hyperbranched mantle, and 3) a corona with reactive surface groups. Dendrimers can be readily synthesized via multistep organic synthesis, through a well‐developed divergent (dendrimers grow outward from a central core) or convergent (dendrimers are synthesized inward from the surface group toward the core) approach.^46^ Their size is classified by “generation,” in which each generation corresponds to a layer of branching units. Besides their unique and controllable architectures, dendrimers possess many other fascinating features, including narrow polydispersity index, high loading ability, and multiple accessible functional groups at the periphery, making them outstanding candidates for therapeutic delivery formulations.^47^ Examples of dendrimers as carriers to deliver drugs, proteins, and DNAs have been highlighted in dendrimer review articles.^46^, ^47^, ^48^

**Figure 4:**
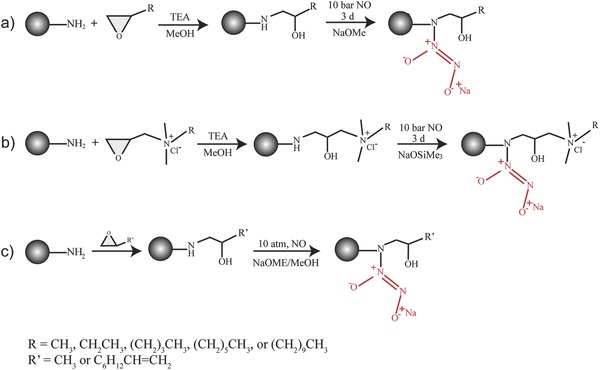
Synthesis of NO‐releasing alkyl‐modified poly(amidoamine) (PAMAM) dendrimers. Terminal primary amines on dendrimers are functionalized to yield secondary amines, which are then treated with NO at high pressure under basic conditions allowing for the formation of *N*‐diazeniumdiolates. a) Reproduced with permission.^50^ Copyright 2016, Elsevier. b) Reproduced with permission.^52^ Copyright 2014, American Chemical Society. c) Reproduced with permission.^53^ Copyright 2013, American Chemical Society.

The Schoenfisch lab, one of the pioneers in the field of NO‐releasing macromolecular scaffolds, reported a protocol to introduce NO moieties onto dendrimers, which involves two sequential steps. First, terminal primary amines on dendrimers are functionalized to yield secondary amines, which are then treated with NO at high pressure under basic conditions allowing for the formation of NONOate (**Figure** **4**).^49^, ^50^, ^51^, ^52^, ^53^, ^54^, ^55^ His group reported that size, type, and exterior functionalities of dendrimers have a significant effect on the NO release kinetics and antimicrobial activities of NO‐releasing dendrimers. NO‐releasing generation 1 (G1) poly(amidoamine) (PAMAM) dendrimers with alkyl chains of varying length (i.e., propyl, butyl, hexyl, octyl, and dodecyl) were synthesized via a ring‐opening reaction with NO payload of ≈1 µmol mg^−1^.^50^ NO release profiles and antimicrobial activities of these systems were investigated at pH 7.4 and 6.4 (associated with dental caries). At lower pH, a faster NO release was observed for all alkyl‐modified G1 PAMAM dendrimer systems, due to the proton‐initiated decomposition of NO donors, leading to ≈4‐fold increase of maximum NO flux ([NO]_max_ = ≈20 000 ppb mg^−1^ at pH 6.4 vs ≈4500 ppb mg^−1^ at pH 7.4). The NO release half‐life increased from 4 to 9 min with longer alkyl chains, likely attributed to a decrease in water diffusion to the NO donors due to the increasing hydrophobicity. The bactericidal and anti‐biofilm efficacy of NO‐releasing alkyl‐modified G1 PAMAM dendrimers was evaluated against planktonic *Streptococcus mutans* (*S. mutans*) or *S. mutans* biofilms, respectively. Octyl‐ and dodecyl‐modified PAMAM dendrimers were the most effective for eradicating *S. mutans* biofilms, which demonstrates the potential of NO‐releasing alkyl‐modified PAMAM dendrimers for the treatment of dental caries. The intercalation of long alkyl chains into bacterial membrane introduced efficient membrane disruption prior to significant intracellular NO accumulation.

To investigate the role of dendrimer size (generation) on NO release kinetics and anti‐biofilm efficacy, NO‐releasing alkyl‐modified G1 to G4 PAMAM dendrimers were synthesized.^51^ All designed dendrimers displayed analogous NO release profiles regardless of dendrimer generation, with NO payload of ≈1 µmol mg^−1^, *t*_1/2_ = 20–30 min, and NO release continued for 7–10 h. The bactericidal activities of these systems, on the other hand, strongly relied on dendrimer size, with a sizable increase in antibacterial action at higher generations. The study was then extended to the development of quarternary ammonium (QA)‐functionalized NO‐releasing G1 and G4 PAMAM dendrimers with different alkyl chains (i.e., methyl, butyl, octyl, and dodecyl).^52^ Regardless of dendrimer generation (G1 or G4), [NO]_max_ decreased with increasing QA alkyl chain length, e.g., from 15 000 ppb mg^−1^ to 5000 ppb mg^−1^. The hydrophobic nature of the dendrimers increased with increasing chain length, thus leading to a decrease in water diffusion to the proton‐labile NO donors.

It has been reported that NO‐releasing dendrimers with hydrophobic motifs improved biocidal behavior but could become aggressive to mammalian cells simultaneously, which impeded their possible applications in the biomedical field. Herein, Lu et al. developed NO‐releasing G1 PAMAM dendrimers with tunable exterior hydrophobicity by adjusting the ratio of propylene oxide (PO) and 1,2‐epoxy‐9‐decene (ED) grafted onto the dendrimers.^53^ All dendrimers depicted comparable NO release profiles (*t*_1/2_ = ≈1 h). The dendrimers with equal PO to ED ratio exhibited the greatest antibacterial activities without compromising biocompatibility to mammalian cells.

G2 and G5 NO‐releasing poly(propylene imine) (PPI) dendrimers with different exterior surroundings (i.e., aromatic styrene oxide (SO), hydrophilic PEG, or hydrophobic PO) were developed by Sun et al.^54^ Larger dendritic scaffolds produced ≈4–6‐fold greater NO flux than their smaller equivalents, suggesting the importance of dendrimer generation in localized delivery of NO. Generally, G5 dendritic systems were more potent in combating *Pseudomonas aeruginosa* (*P. aeruginosa*) and *Streptococcus aureus* (*S. aureus*) than the corresponding G2 dendrimers, as evidenced by the remarkable decrease in concentration required to eradicate the bacteria strains tested. For each dendrimer generation, SO‐modified NO‐releasing dendrimers showed the lowest minimum bactericidal concentration (MBC) value, while PEG‐functionalized NO‐releasing dendrimers displayed the highest. The different bactericidal activity is due to the surface charge of the dendrimers. Positively charged SO‐modified PPI dendrimers facilitated electrostatic interaction with the bacteria and enhanced antibacterial efficacy. Among all prepared dendrimers, G2 and G5 NO‐releasing dendrimers with SO groups stand out because they maintained minimal toxicity against L929 fibroblast cells even at concentrations needed to achieve 5‐log bacterial killing effect.

### 3.4 Silica and Gold Nanoparticles

**Figure 5:**
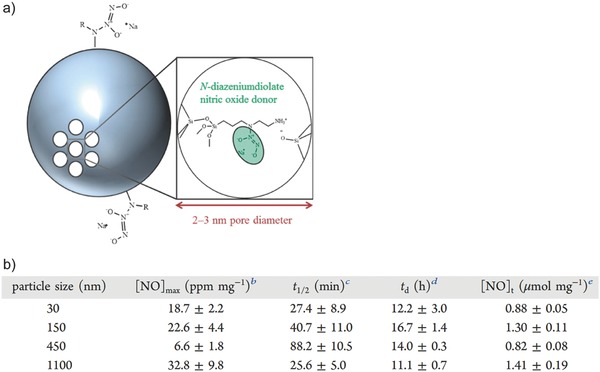
a) Schematic illustration of NO‐releasing mesoporous silica nanoparticles where *N*‐diazeniumdiolate NO donors are formed at the secondary amines. b) NO release properties as a function of size of the mesoporous silica nanoparticles. Reproduced with permission.^59^ Copyright 2016, American Chemical Society.

Easy fabrication, excellent biocompatibility, and flexible surface chemistry have made silica nanoparticles one of the most used carriers to achieve localized delivery.^56^, ^57^, ^58^ Recently, Soto et al. reported the preparation of NO‐releasing mesoporous silica nanoparticles (MSNs) ranging from 30 to 1100 nm through an aminosilane‐template surfactant ion exchange reaction (**Figure** **5**a).^59^ Aminosilane‐modified MSNs were reacted with NO gas under basic conditions yielding encapsulation of NONOates within the pore network. NO payload was reported to be ≈1 µmol mg^−1^, similar to the abovementioned NO‐releasing dendrimers. [NO]_max_ and *t*_1/2_ were found to be significantly different across the four prepared NO‐releasing systems (30, 150, 450, and 1100 nm). [NO]_max_ was the highest for the largest MSNs (33 ppm mg^−1^ for 1100 nm MSNs), while half‐lives ranged from 25 to 88 min, with duration of NO release ranging from 11 to 17 h (Figure 5b). The particle size and position of NO donors within the MSN network affected the rate of water access to the NO donors, leading to tunable NO release kinetics.

Physicochemical properties of silica nanoparticles (size and shape) as carriers for NO donors play an important role in killing bacterial biofilms.^60^, ^61^, ^62^ NO‐releasing silica nanoparticles with identical NO storage (≈0.3 µmol mg^−1^) but different sizes (14, 50, and 150 nm) were fabricated to assess the effect of size in Gram‐negative *P. aeruginosa* and Gram‐positive *S. aureus* biofilm killing capability.^60^ Values of MBC proved that smaller particles (14 nm) were more potent than the larger ones in killing both *P. aeruginosa* and *S. aureus* biofilms. To inspect the effect as a function of particle shape, NO‐releasing nanoparticles with varied aspect ratios (AR1, AR4, and AR8) were produced.^61^ NO storage was consistent among the three types of particles (≈0.7 µmol mg^−1^). The more spherical NO‐releasing particles (AR1) were found to be less effective against both bacterial biofilms compared to rod‐like particles (AR4 and AR8), indicating that higher aspect ratio was conducive in improving biofilm eradication efficacy. In conclusion, smaller sizes and higher aspect ratios are the most effective in biofilm eradication.

Surface chemistry of silica nanoparticles also plays a role in their NO‐releasing properties.^61^, ^63^ Modification of AR4 silica nanoparticles with PEG groups led to shorter *t*_1/2_ (≈10 min) compared to the pristine particles due to a more rapid water uptake by hydrophilic PEG chains on the surface of the particles, leading to faster decomposition of NO donors.^61^ Bactericidal NO dose for PEG‐functionalized AR4 silica nanoparticles was approximately half of that for pristine AR4 silica nanoparticles, suggesting that greater NO flux is favorable for bacteria killing.

Gold nanoparticles (AuNPs) with core sizes from 1 to 100 nm serve as a highly multifunctional theranostic platform owing to their unique physical, chemical, optical, and electronic properties.^64^, ^65^, ^66^ Boyer and co‐workers reported NO‐releasing AuNPs by surface functionalization of the particles with hexylamine‐modified poly(oligoethylene glycol methyl ether methacrylate)‐*b*‐poly(vinyl benzyl chloride) (P(OEGMA)‐*b*‐PVBHA)), followed by postmodification to facilitate the formation of NONOates.^67^ The hybrid particles continued to release NO via protonation‐induced decomposition over 6 d at pH 6.8 and ambient temperature, and were shown to be effective in two different applications, *P. aeruginosa* biofilm dispersal and cancer cell cytotoxicity. While tremendous advances in therapeutic AuNP technologies have been developed, there are challenges associated with their clinical translation. Although AuNPs are inherently nontoxic, they are not biodegradable, which may lead to plausible toxicological effects derived from accumulation of non‐biodegradable nanoparticles in the body. A number of studies suggested that AuNPs could activate immune cells^68^, ^69^ and as with other nanoparticle formulations, controlling passage of AuNPs across biological barriers is not trivial.

### 3.5 Polymer Particles

**Figure 6:**
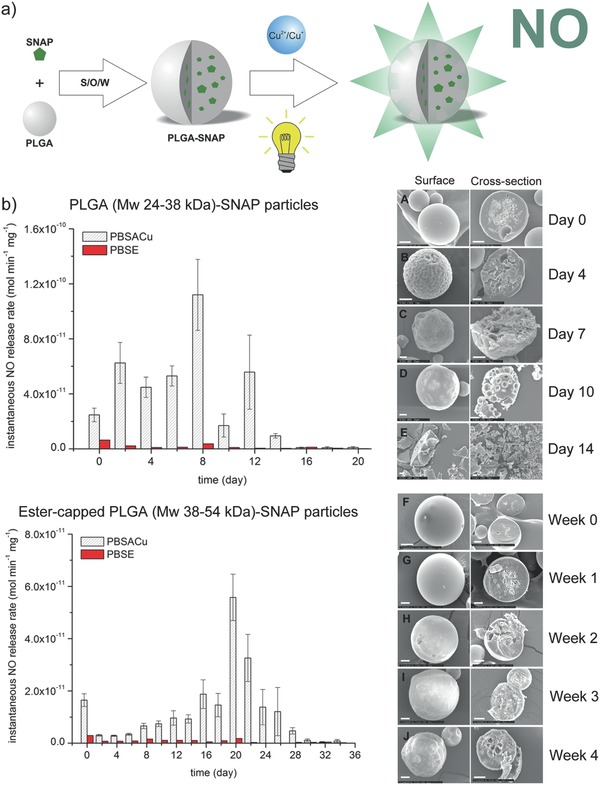
a) Schematic illustration of the formation of *S*‐nitroso‐*N*‐acetylpenicillamine (SNAP)‐poly(lactic‐*co*‐glycolic‐acid) (PLGA) particles using a solid‐in‐oil‐in‐water emulsion solvent evaporation method and approaches to trigger NO release from these particles. b) Left: NO release rate of pristine PLGA‐SNAP microspheres (top) and ester‐capped PLGA‐SNAP microspheres (bottom) in PBS containing copper ions and ascorbic acid (PBSACu) or EDTA (PBSE). Right: Secondary electron micrographs of the surface and the cross‐section of PLGA‐SNAP microspheres (top) and ester‐capped PLGA‐SNAP microspheres (bottom) over time. Scale bar: 20 µm. Reproduced with permission.^72^ Copyright 2016, Elsevier.

Poly(lactic‐*co*‐glycolic‐acid) (PLGA) particles have been extensively used as drug delivery carriers due to their biocompatibility and biodegradability properties.^70^, ^71^ PLGA is an FDA approved polymer with a long clinical experience and can be employed for sustained drug release (months). Meyerhoff and Schwendeman encapsulated NO donor SNAP within PLGA microspheres using a solid‐in‐oil‐in‐water emulsion solvent evaporation method (**Figure** **6**a).^72^ SNAP generates NO through: 1) copper‐ion mediated decomposition, 2) reactions with ascorbate, and 3) homolytic cleavage of the S‐NO bond by light. Different polymer molecular weight and surface modification of the particles were used to modulate the NO release profile. In the presence of copper(II) ions and ascorbic acid, release of NO continued for over 10 d for pristine PLGA (*M*_w_ = 24 000–38 000) particles, while an extended NO release (four weeks) was observed when ester‐capped PLGA (*M*_w_ = 38 000–54 000) microspheres were applied (Figure 6b). Higher molecular weight, together with the ester terminus, slowed the bioerosion of ester‐capped PLGA and hence prolonged the liberation of NO. Rapid release of NO (within 6 h) can be induced via light irradiation. This platform enables tunability of NO release from hours to one month from the same biomaterials. The SNAP/PLGA particles were stable when stored at room temperature for up to one year.

Yoo and co‐workers encapsulated a different type of NO donor (polyethylenimine (PEI)/NONOate) into PLGA particles.^73^ PEI/NONOate was synthesized by reacting NO with secondary amine groups of PEI and reported to contain 1.4 µmol of NO per mg of polymer. Incorporation of PEI/NONOate into PLGA nanoparticles led to a sustained release of NO by hydrolysis over 6 d (compared to 12 h for nonencapsulated PEI/NONOate). This enhancement was expected since the hydrophobic PLGA matrix strongly restricted water diffusion and thus slowed the degradation of NONOate moieties. This class of NO‐releasing PLGA particles induced more than 4‐log and 3‐log reduction in bacterial viability against methicillin‐resistant *S. aureus* and *P. aeruginosa*, respectively, at a concentration of 10 mg mL^−1^. The electrostatic interactions between positively charged PEI/NONOate‐PLGA particles (+35 mV) and bacterial surfaces assisted the direct delivery of NO to sites of interest. PEI/NONOate‐PLGA particles at a concentration of 5 mg mL^−1^, however, were observed to induce a 35% reduction in fibroblast viability. Therefore, further investigation on mitigating the cytotoxicity of PEI/NONOate‐PLGA particles is required.

**Figure 7:**
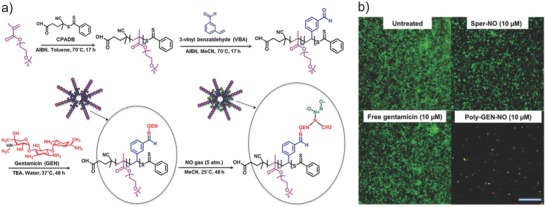
a) Synthesis of gentamicin‐NONOate polymeric nanoparticles. Gentamicin is firstly conjugated to pre‐synthesized poly(oligoethylene glycol methyl ether methacrylate)‐*b*‐poly(3‐vinylbenzaldehyde) (POEGMA‐*b*‐PVBA) nanoparticles. Gentamicin has both primary and secondary amine groups, and in the next step, NO donor is directly formed by the reaction of gentamicin with NO gas to yield a unique gentamicin‐NONOate complex. b) Confocal microscope images of *Pseudomonas aeruginosa* biofilms stained with LIVE/DEAD kit. Biofilms are treated with NO donor spermine NONOate (Sper‐NO), free gentamicin, or gentamicin‐NONOate polymeric nanoparticles (Poly‐GEN‐NO). The gentamicin‐NONOate nanoparticles strongly kill bacterial biofilm compared to those inoculated with NO donors or gentamicin alone. Scale bar: 50 µm. Reproduced with permission.^74^ Copyright 2015, The Royal Society of Chemistry.

The Boyer and co‐workers reported codelivery of NO and antibiotic gentamicin using polymeric nanoparticles to effectively disperse *P. aeruginosa* biofilms.^74^ Gentamicin was first conjugated to presynthesized poly(oligoethylene glycol methyl ether methacrylate)‐*b*‐poly(3‐vinylbenzaldehyde) nanoparticles. Gentamicin have both primary and secondary amine groups, and in the next step, NO donor was directly formed by the reaction of gentamicin with NO gas to yield a unique gentamicin‐NONOate complex (**Figure** **7**a). The gentamicin‐NONOate nanoparticles strongly decreased the viability of both biofilm and planktonic cells compared to those inoculated with NO donors or gentamicin alone (Figure 7b), suggesting the synergistic effect between NO and gentamicin.

### 3.6 Metal–Organic Frameworks

Metal–organic frameworks (MOFs) are crystalline structures made of organic linkers bound to metal centers. They represent an emerging class of porous materials (pore size in the range of 0.3–6 nm), with extremely high surface area (can be up to 14 600 m^2^ g^−1^).^75^, ^76^, ^77^ MOF properties can be tuned by a number of possible metal center‐organic linkers, and the combination of high surface area and tunable pore size lead to a growing interest in MOF as a storage and delivery system for NO. Generally, NO is adsorbed (chemisorbed or physisorbed) within the pores. Lowe et al. reported NO‐releasing Cu‐TDPAT, a class of MOF that contains copper nodes linked by 2,4,6‐tris(3,5‐dicarboxylphenylamino)‐1,3,5‐triazine (H_6_TDPAT).^78^ NO loading was achieved via two approaches: 1) NO adsorption in the pores and 2) the reaction of NO with secondary amine groups of the TDPAT linkers to form NONOates. The MOF containing NO was placed in a chamber that maintained N_2_ at 85% relative humidity, in which water reacted with the sample to release NO from the Cu‐TDPAT framework. Weakly adsorbed NO in the pores of the Cu‐TDPAT was released in ≈40 min, while the covalently bound NONOates allowed for greater control over the NO delivery rate. Over the course of 7 d, the Cu‐TDPAT released 175 µmol of NO per gram of Cu‐TDPAT. An iron(II)‐based MOF, Fe_2_(dobdc) (dobdc^4−^ = 2,5‐dioxido‐1,4‐benzenedicarboxylate), is one of the newly discovered MOFs that possess both high density of coordinatively unsaturated sites and the biocompatibility of iron. Bloch et al. reported Fe_2_(dobdc) with an NO storage ability of 6.5 mmol g^−1^ and a biphasic NO release profile was observed when the MOF containing NO was exposed to N_2_ at 11% relative humidity at 37 °C.^79^ An initial burst release within the first 3 d was observed from weakly adsorbed NO, followed by a slow NO release over 7 d likely due to structural rearrangement or the partial collapse of Fe_2_(NO)_2_(dobdc).

**Figure 8:**
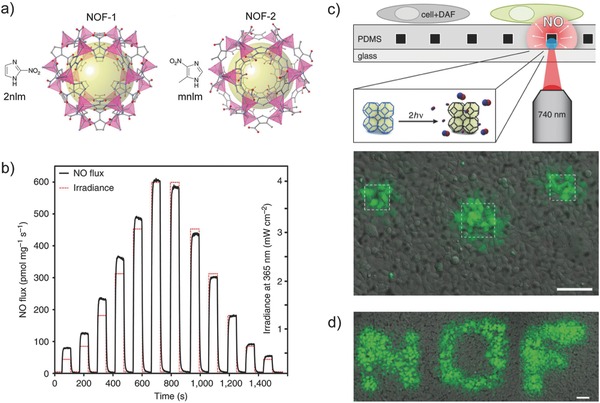
a) Schematic illustration of photoresponsive NO‐releasing metal–organic frameworks (MOFs): Zn(2nIm)_2_ (NOF‐1) and Zn(mnIm)_2_ (NOF‐2). b) NO release profile of NOF‐1 can be tuned by varying light irradiation time and intensity. c) Top: Schematic illustration of localized NO release from NOF‐1 triggered by two‐photon near‐infrared laser irradiation. The released NO reacts with NO fluorescent indicator, 4‐amino‐5‐methylamino‐2′,7′‐difluorofluorescein (DAF‐FM), generating green fluorescent signal. Bottom: Confocal microscope image demonstrating the selective photoactivation of NOF‐1 on human embryonic kidney 293 (HEK293) cell culture. Scale bar: 100 µm. d) Further demonstration of spatiotemporal control activation of NOF‐1. Scale bar: 100 µm. Reproduced with permission.^80^ Copyright 2013, Nature Publishing Group.

The MOF systems described above released NO by displacement or through reaction with water molecules. The Kamei and Furukawa lab reported an interesting photoresponsive NO‐releasing MOF that enabled precise controlled NO delivery at the cellular level triggered via localized two‐photon laser activation.^80^ In their study, photoactive NO donors based on nitroaromatic compounds, 2‐nitroimidazole (2nIm) and 5‐methyl‐4‐nitroimidazole (mnIm), were incorporated in a zeolitic imidazolate framework (ZIF) to form Zn(2nIm)_2_ (NOF‐1) and Zn(mnIm)_2_ (NOF‐2), respectively (**Figure** **8**a). NOF‐1 and NOF‐2 did not release any NO molecules under ambient conditions, but did release 3.4 and 2.9 µmol mg^−1^ of NO, respectively, upon a 3 h continuous light irradiation. The NO flux produced can be simply tuned by controlling irradiation time and intensity (Figure 8b). To demonstrate the spatiotemporal NO delivery from NOFs in a cellular environment, NOF‐1 microcrystals were spin‐cast on a glass‐bottomed dish, followed by deposition of a polydimethylsiloxane (PDMS) layer via spin‐coating. The NOF‐1/PDMS were coated with Matrigel to facilitate cell adhesion and then human embryonic kidney 293 (HEK293) cells were cultured on the substrate. The localized photoactivation of NOF‐1 led to the release of NO, which reacted with an intracellular NO fluorescent indicator, 4‐amino‐5‐methylamino‐2′,7′‐difluorofluorescein diacetate (DAF‐FM DA), and induced a fluorescent response in the surrounding cells (Figure 8c). Further demonstration of spatiotemporal control was shown by writing “NOF” upon activation of the selected regions (Figure 8d).

## 4 NO Delivery from Implantable Materials

**Figure 9:**
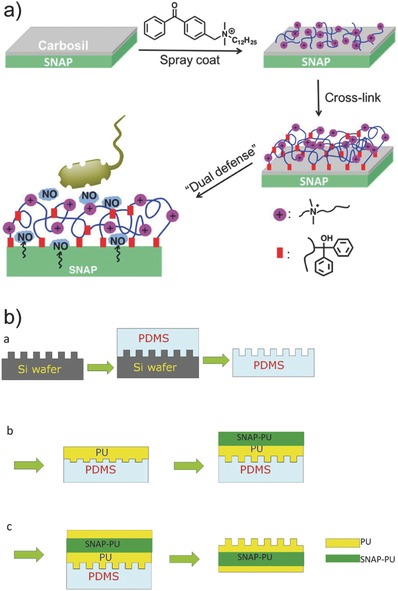
a) Fabrication of *S*‐nitroso‐*N*‐acetylpenicillamine (SNAP)‐benzophenone based quaternary ammonium (BPAM) films. SNAP is incorporated in CarboSil polymer, followed by the sequential immobilization of antimicrobial molecules, BPAM, onto the top of the SNAP‐CarboSil films. Reproduced with permission.^82^ Copyright 2017, Elsevier. b) Process to fabricate a sandwich film, consisting a textured top polyurethane (PU) layer, a SNAP‐doped middle PU layer, and a bottom smooth PU layer via a soft lithography two‐stage replication molding technique. Reproduced with permission.^83^ Copyright 2017, Elsevier.

Polymer films are a great tool of delivery system as they can maintain a high local concentration of therapeutic agent at a specific site.^81^ Freestanding drug‐containing polymer films can be directly attached on the pathological site of tissues or they can be used as coatings deposited on medical devices such as implants or stents. Implantable medical devices are critical in restoring body functions, however their performance can be affected by undesirable blood clot formation and bacterial infection. Films or coatings containing NO with inherent antithrombotic and antibacterial properties offer a solution to these issues. The development of NO‐releasing polymer films with multiple antibacterial mechanisms was demonstrated by Pant et al. These films were developed through incorporation of NO donor SNAP in CarboSil polymer and sequential immobilization of antimicrobial molecules (benzophenone based quaternary ammonium (BPAM)) on top of the SNAP‐CarboSil films (**Figure** **9**a).^82^ Compared to pristine SNAP films, an increase in NO flux was observed for SNAP‐BPAM films over a 24 h period. This is not surprising since the presence of positively charged ammonium functional groups on BPAM topcoats increased the film hydrophilicity. Dual‐action SNAP‐BPAM films showed a 4‐log reduction in bacterial viability for *S. aureus* biofilms and a 3‐log reduction for *P. aeruginosa* biofilms, compared to control CarboSil films. On the other hand, single‐action BPAM films reduced the viability of *S. aureus* by 3 log units and single‐action SNAP films reduced the viability of *P. aeruginosa* by 2 log units. These data clearly implied that the combination of SNAP and BPAM is favorable in eradicating bacterial biofilms.

NO‐releasing polymer films with a sandwich‐like architecture, consisting a textured top polyurethane (PU) layer, a SNAP‐doped middle PU layer, and a bottom smooth PU layer, was reported by Xu et al. (Figure 9b).^83^ The NO release profile and bactericidal activities of these films were investigated as a function of the amount of SNAP doped (5, 10, 15 wt%) and texture patterns. Compared to regular smooth PU surfaces, textured patterns greatly increased film hydrophobicity due to air captured in the spaces between pillars, as reflected by the larger corresponding water contact angle of 139°. The NO release lasted for days at an NO flux >0.05 nmol min^−1^ cm^−2^, corresponding to the level produced by healthy endothelial cells. Compared to films with smooth top layers, SNAP films with textured surfaces reduced *S. epidermidis* bacterial adhesion and inhibited biofilm formation for over 28 d.

**Figure 10:**
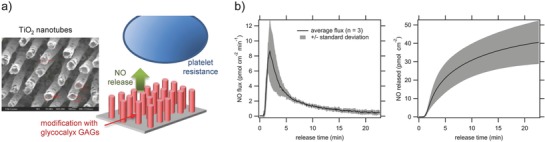
a) Schematic illustration of NO‐releasing titanium dioxide nanotube (TiO_2_NT)‐based films that resemble the morphological features of endothelial glycocalyx, and scanning electron microscopy image of the TiO_2_ nanotubes (left). b) NO flux and cumulative NO release from the TiO_2_NT+NO multilayer surface. Reproduced with permission.^86^ Copyright 2016, American Chemical Society.

The luminal surface of healthy blood vessels prevents inflammation and coagulation while in continuous contact with flowing blood. They are able to maintain these functions due to the presence of a glycosaminoglycan‐rich brush‐like layer called glycocalyx, which serves as a mechanosensor of the endothelium that is responsive to shear‐induced NO production.^84^, ^85^ Simon‐Walker et al. synthesized NO‐releasing titanium dioxide nanotube (TiO_2_NT)‐based films that resemble the morphological features of the endothelial glycocalyx (**Figure** **10**a).^86^ TiO_2_NTs were coated with heparin/nitrosated‐chitosan polyelectrolyte multilayers (PEMs) via a layer‐by‐layer assembly process. TiO_2_NT+PEM+NO released 40 pmol cm^−2^ NO over 20 min, suggesting a relatively rapid NO release from this bioinspired structure (Figure 10b). When cocultured with whole blood plasma containing platelets, significant differences in cell adhesion were observed for TiO_2_NT+PEM+NO compared to TiO_2_NT and TiO_2_NT+PEM, despite the presence of chitosan that enhanced cell adhesion. TiO_2_NT+PEM+NO decreased platelet aggregation and activation, as reflected by the small number of platelets presented with conserved round shapes. This study showed that collectively, factors such as NO releasing ability, surface topography, and chemistry are crucial in achieving antithrombotic activities.

**Figure 11:**
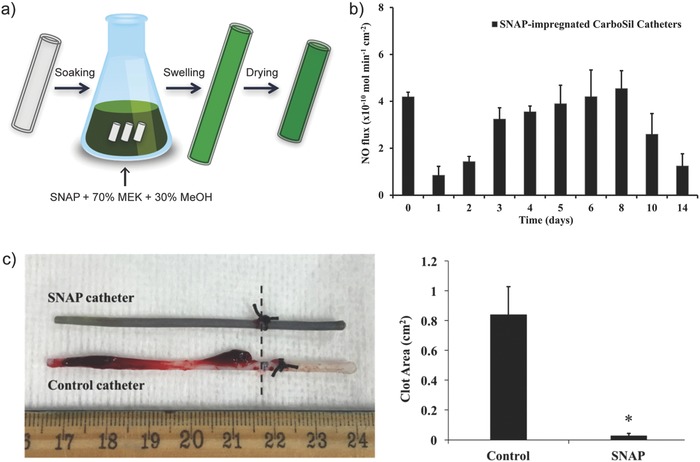
a) Preparation of *S*‐nitroso‐*N*‐acetylpenicillamine (SNAP)‐impregnated CarboSil intravascular catheters. The catheter is soaked in a SNAP solution in 70% methyl ethyl ketone (MEK) and 30% methanol (MeOH). b) NO release profile of the SNAP‐impregnated catheter at physiological conditions over time. c) Photo showing a decrease in thrombus formation on a SNAP‐impregnated CarboSil catheter compared to control CarboSil catheter after 7 h implantation in rabbit external jugular veins, and the corresponding measured clot area. Reproduced with permission.^87^ Copyright 2017, American Chemical Society.

**Figure 12:**
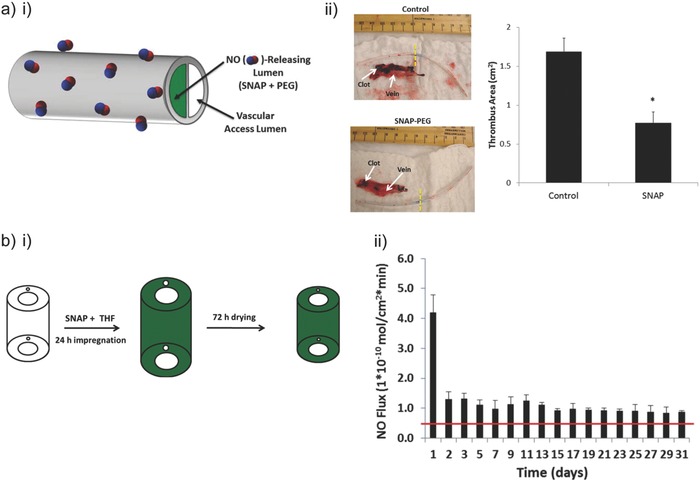
a) i) Dual lumen intravascular catheters, where one lumen is filled with *S*‐nitroso‐*N*‐acetylpenicillamine (SNAP) and poly(ethylene glycol) (PEG) and another lumen for vascular access. ii) Photos showing a decrease in thrombus formation on a SNAP‐PEG catheter compared to control PEG catheter after 11 d implantation in rabbit veins, and the corresponding measured thrombus area. Reproduced with permission.^89^ Copyright 2016, American Chemical Society. b) i) Preparation of NO‐releasing silicone Foley catheters where SNAP is impregnated via a solvent‐swelling method. ii) NO release profile of the SNAP‐impregnated silicone Foley catheter at physiological conditions over time. Reproduced with permission.^90^ Copyright 2015, American Chemical Society.

Wo et al. reported the application of NO‐releasing coating onto CarboSil intravascular catheters (**Figure** **11**a).^87^ Catheters were soaked in a SNAP solution, followed by coating with a CarboSil polymer solution. The coated catheters released NO by crystal (SNAP) dissolution process when submerged in 10 × 10^−3^ m PBS containing 100 × 10^−6^ m EDTA (pH 7.4, 37 °C). A unique NO release trend was observed for catheters containing 15 wt% SNAP: initial burst release (≈0.4 nmol min^−1^ cm^−2^) within 24 h due to the rapid decomposition of SNAP from the outermost layer of the catheter surface; after depletion of the SNAP reservoir in the outermost region, the NO release rate dropped to its minimal level on day 1 (≈0.1 nmol min^−1^ cm^−2^) and then gradually increased over the next 8 d (Figure 11b). The NO release levels then dropped to below 0.05 nmol min^−1^ cm^−2^ after day 14. At this point all the SNAP in the bulk of the polymer had decomposed. The majority of the SNAP molecules impregnated in the catheters were in their crystalline form, and it took time for the crystalline SNAP embedded in the bulk of the polymer to dissolve and release its NO. The number of viable *S. epidermidis* and *P. aeruginosa* adhered on the surface of SNAP‐impregnated catheter segments after 14 d was reduced by 2 and 2.5 log units, respectively, demonstrating that continuous NO release may lead to reduced risk of catheter‐related bloodstream infections. In vivo experiments using an acute 7 h rabbit thrombogenicity model were conducted to examine the effect of the SNAP‐impregnated catheters on decreasing clot formation. The clot area on control catheter was calculated to be 0.84 cm^2^, whereas the clot area on the SNAP catheter was 0.03 cm^2^ (Figure 11c). A similar coating principle was applied to fabricate NO‐releasing central venous catheters, in which coatings consisted of Elast‐eon E2As polymer with diazeniumdiolated dibutylhexanediamine (DBHD/NONO) and PLGA additives.^88^ NO release was modulated via the hydrolysis rate of the PLGA. Another report demonstrated a modification of commercially available dual lumen intravascular catheters, by filling one lumen with a composite mixture of PEG and SNAP (with different PEG molecular weights and SNAP concentrations), leaving another lumen for vascular access (**Figure** **12**ai).^89^ Antibacterial activity of PEG‐SNAP catheters was assessed using an in vitro CDC bioreactor. Over 98% reduction in bacterial (*E. coli* and *S. aureus*) viability was observed on the surfaces of PEG (*M*_w_ 4 000)‐SNAP (40 wt%) catheters compared to the corresponding controls. After 11 d implantation in rabbit veins, thrombus formation on the PEG‐SNAP catheters was significantly less than observed for the PEG control catheters (0.77 vs 1.70 cm^2^, respectively) (Figure 12aii).

To reduce the associated notorious urinary tract infection caused by certain microbes (*S. epidermidis* and *Proteus mirabilis*) when using urinary catheters, commercial silicone Foley catheters were impregnated with SNAP by a simple solvent‐swelling approach (Figure 12bi).^90^ At physiological conditions (37 °C, pH 7.4), the functionalized catheters continued to release NO for at least 30 d with NO flux ranging from 0.08–0.14 nmol min^−1^ cm^−2^ (Figure 12bi). NO release kinetics of the modified catheters at pH 6.5 and 8.0 was similar to that at pH 7.4. A 3.7‐log reduction in *S. epidermidis* and a 6‐log reduction in *P. mirabilis* viability were achieved using SNAP‐impregnated catheters over 14 d.

## 5 NO Delivery via Catalytic Approaches

An approach that draws inspiration directly from nature is to generate, synthesize NO specifically where and when it is needed. Radical NO species are short‐lived. For this reason, controlled delivery of precise amounts of NO with spatiotemporal resolution is highly challenging. Even for the fine‐tuned systems where such control is exerted, reservoir‐type depots are disadvantages in terms of the deliverable payload by the finite pool of the NO donor. Nature offers a straightforward approach to deal with these problems—to perform localized synthesis of NO. Several isoforms of NOS enzymes are known, most notably nNOS, eNOS, and iNOS types.^91^ Enzymatic synthesis of NO is highly localized and can generate continuous and steady amounts of the product. It relies on endogenous, nutritional precursors such as l‐arginine and is therefore “limitless” in terms of the deliverable payload. Enzymatic output and the amount of NO generated is tightly controlled and can be up/downregulated, specifically through variation of concentration of the NOS cofactors (nicotinamide adenine dinucleotide phosphate, flavin adenine dinucleotide, flavin mononucleotide, and (6R‐)5,6,7,8‐tetrahydrobiopterin (BH4)).^91^ These attributes of a localized pool of NO are highly appealing and inspired engineering of synthetic implantable biomaterials with capacity to achieve localized synthesis of NO. Two strategies emerge from the knowledge of natural, enzymatic NO synthesis: 1) implementation of “enzyme‐prodrug therapy” (EPT) based on natural enzymes and custom‐made synthetic prodrugs of NO, and 2) design of enzyme mimics that produce NO using endogenous donors. These developments are quite recent, with the first reports dating back by only two decades and a considerable surge of interest observed in the last few years (judging by the number of publications on the subject).

### 5.1 Localized Synthesis of NO Using Natural Enzymes and Synthetic Prodrugs

Development of enzyme‐prodrug pairs for the synthesis of NO is encouraged by the success of EPT, a suit of techniques specifically developed for the localized synthesis of drugs using externally added, benign prodrugs.^92^ Conceived in mid‐1980s, development of EPT primarily focuses on the methods of immobilization of the enzyme which then performs the bioconversion of prodrugs at the site where the drug needs to be synthesized, most commonly cancerous tissue. This can be achieved via surgical placement of the enzyme or the enzyme‐containing biomaterial,^93^, ^94^, ^95^ using polymers and liposomes,^96^ antibodies,^97^ bacteria,^98^ mammalian cells,^99^ and/or cell mimics.^100^ Success of EPT also in large part depends on the choice of the prodrug.^101^ Of all of the drug candidates, NO appears to be highly deserving for the implementation of EPT, specifically because of its short half‐life. Indeed, NO release upon enzymatic activation of the prodrugs has been engineered to be triggered by cytochromes,^102^ esterase,^103^ oxireductase,^104^ nitroreductase,^105^ and glycosidase.^106^, ^107^ Much of the credit for these developments goes to Keefer et al.^108^ who were most active in the field. In recent years, much focus was placed on β‐galactosidase (β‐gal) as an enzyme to trigger the release of NO from the corresponding prodrug β‐gal‐NONOate with several teams reporting successful examples of the systems engineered around this enzyme‐prodrug pair.^109^, ^110^, ^111^, ^112^ Another important development concerns glycosylated prodrugs that are developed for targeted drug delivery. *N*‐acetylglucosamine‐containing NONOates were used to achieve an efficient uptake by macrophages through the mannose receptor which resulted in an intracellular release of NO and ensuing activity against an intracellular protozoan parasite (*Leishmania major*).^113^

The overall majority of efforts in EPT for delivery of NO rely on the intracellular activation of the prodrugs. This comes in stark contrast with the EPT methods for delivery of typical anticancer drugs, in which case prodrug bioconversion is typically pursued in the extracellular space to avoid the limitations exerted by the prodrug cell entry. What remains similar is that most of the above‐cited studies, as is the case with the majority of EPT reports, pursue delivery of NO to combat cancer. Notable exception is the intracellular delivery of NO as a measure to treat intracellular parasites.^113^

**Figure 13:**
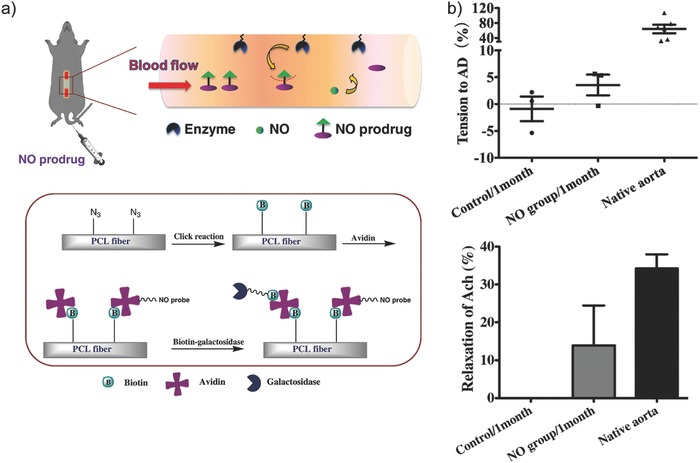
a) Top: Schematic illustration of the catalytic activities of enzymes (galactosidase) immobilized on poly(ε‐caprolactone) (PCL) vascular grafts to liberate NO from externally administered NO prodrugs. Bottom: Surface functionalization of galactosidase onto PCL vascular grafts via biotin–avidin interaction. b) Contractile response of explanted vascular grafts to adrenaline (AD) (top) and relaxation response to acetylcholine (Ach) (bottom) compared to native aortic functions. Reproduced with permission.^114^ Copyright 2015, Elsevier.

Here, we highlight a number of recent reports on controlled delivery of NO using β‐gal/β‐gal‐NONOate enzyme‐prodrug pair. Wang et al. engineered enzymatic release of NO using β‐gal on the surface of model vascular grafts.^114^ NO is recognized as a “guardian of vascular grafts”^115^ in that this molecule spells anti‐adhesion signals to circulating platelets, antiproliferative stimulus to myoblasts, and positive proliferation stimulus to endothelial cells. Vascular grafts with engineered localized synthesis of NO were successful in vivo demonstrating decreased platelet adhesion and enhanced establishment of endothelial lining.^114^ Most impressively, explanted vascular grafts exhibited native (albeit decreased) aortic functions on the myograph, namely increase in tension in response to adrenaline and decrease in tension in response to acetylcholine (**Figure** **13**). In a recent report, Li and co‐workers developed NO‐releasing hydrogels as a platform to successfully direct endothelial differentiation of mouse embryonic stem cells without the need to add exogenous growth factors.^116^ β‐gal/β‐gal‐NONOate pair was used to release NO in a controllable and sustainable manner for more than 48 h. To date, the source of endothelial cells for transplantation for treating various kinds of vascular diseases is limited due to the lack of proper environment for generating therapeutic endothelial cells in vitro. Therefore, the ability to generate high quality and sufficient amount of therapeutic endothelial cells represents a significant promise in the field of cell therapy.

**Figure 14:**
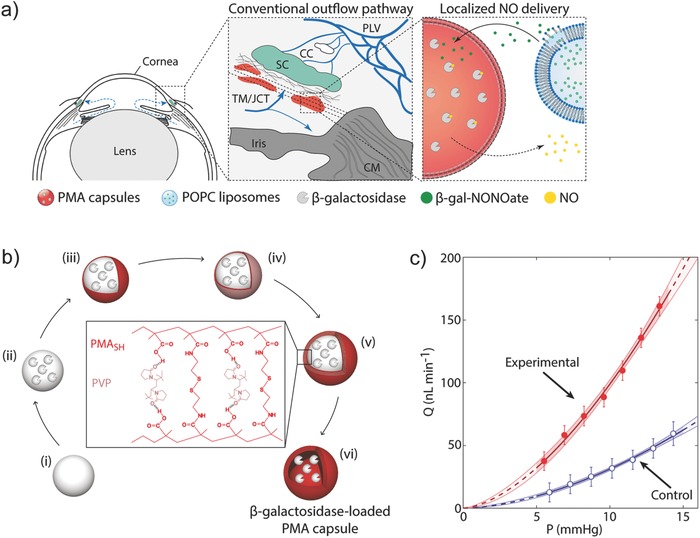
a) Localized delivery of NO to the conventional outflow pathway via enzyme‐prodrug therapy, employing β‐galactosidase‐loaded polymer capsules and β‐gal‐NONOate‐loaded liposomes. b) Schematic illustration of assembly of β‐galactosidase‐loaded polymer capsules through a sequential deposition of thiolated poly(methacrylic acid) (PMA_SH_) and poly(*N*‐vinylpyrrolidone) (PVP). c) Effects of local NO delivery on conventional outflow facility demonstrated in a mouse model. Representative flow‐pressure relationship for a given pair of eyes, indicating an apparent increasing effect of NO delivery on the flow rate inside the eye. Reproduced with permission.^117^ Copyright 2017, Wiley‐VCH.

In our own work, we established a localized bioconversion of β‐gal‐NONOate to NO as a suggested treatment for glaucoma.^117^ NO has a vasodilating effect and when generated locally within the trabecular meshwork, it aids in lowering intraocular pressure and in the conventional outflow of humor in the eye. Immobilization of β‐gal in our work was achieved using micrometer‐sized hydrogel capsules assembled via the sequential polymer deposition technique.^118^, ^119^ In turn, the prodrug was administered using liposomes as depot vessels. We demonstrated that NO delivery using EPT is an on‐demand approach and is initiated when needed by external administration of the prodrug. NO dosing was controlled by the concentration of administered NO donor. The method proved to be efficacious in alleviating elevated intraocular pressure (ex vivo) (**Figure** **14**).

### 5.2 Enzyme Mimics for Conversion of Endogenous Prodrugs of NO

An exciting, highly promising opportunity to achieve localized synthesis of NO is to make use of the natural prodrugs of NO, specifically RSNOs. The latter include GSNO, *S*‐nitrosocysteine (CysNO), and most notably *S*‐nitrosoalbumin (AlbSNO) in blood.^120^ RSNOs are formed via a reaction between endothelium‐derived NO and endogenous thiols, specifically albumin Cys‐34, giving rise to a micromolar natural pool of RSNO.^121^ This endogenous pool presents a life‐long supply of the prodrug and much recent activity has been devoted to the identification of enzymes and more so enzyme mimics to create biomaterials for localized, continuous synthesis of NO via catalytic conversion of RSNO.

**Figure 15:**
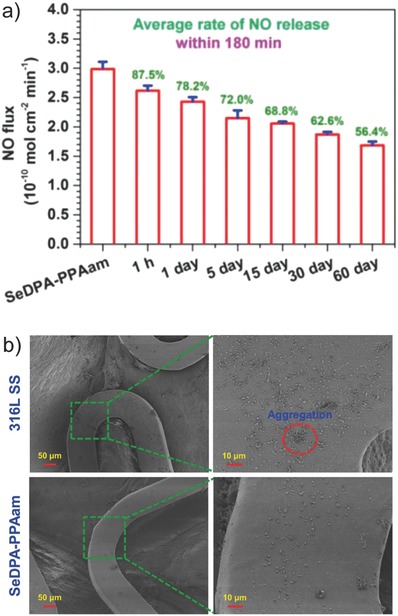
a) NO release from 316L stainless steel coronary artery stents coated with organoselenium SeDPA in the presence of NO donors, with the catalytic performance maintained for at least 60 d. Values in green represent the retention rates of the total amount of NO generated from the SeDPA coating. b) Scanning electron microscopy images of control and SeDPA‐coated stents after implantation into iliac arteries of a rabbit, showing significantly improved anti‐coagulant property of the coated stent. Reproduced with permission.^125^ Copyright 2015, Elsevier.

NO release from RSNOs can also be initiated by glutathione peroxidase, a selenium‐containing enzyme. Based on this knowledge, Cha and Meyerhoff hypothesized that nonproteinaceous organoselenium compounds would be capable of degrading RSNOs and thus be suited for localized synthesis of NO.^122^ This approach proved to be highly successful and was reported in multiple studies by several groups.^122^, ^123^, ^124^, ^125^ Target application for such materials is cardiovascular stenting whereby locally produced NO stimulates endothelial proliferation and decreases platelet adhesion and myoblast proliferation. For example, Weng et al. deposited selenocystamine on polydopamine‐grafted 316L stainless steel coronary stents.^123^ The coated stents catalyzed the decomposition of endogenous RSNOs in vivo and after two month implantation in adult dogs, vascular smooth muscle cell proliferation was significantly inhibited compared to bare stents. This approach has the potential to reduce the incidence of neointimal hyperplasia (thickening of arterial walls). Another study worthy of note is the report from Yang et al. who engineered NO‐catalytic bioactive implant through covalent immobilization of organoselenium compounds on a metal stent surface.^125^ 316L stainless steel coronary artery stents were first functionalized with a plasma polymerized allylamine (PPAam) coating, which provided primary amine groups for coupling organoselenium SeDPA using carbodiimide chemistry. In the presence of NO donors, NO flux of ≈0.3 nmol min^−1^ cm^−2^ was achieved, which is at the level produced by healthy endothelium.^6^, ^7^ Clear benefits of these materials is that these are not biodegradable and can maintain their catalytic performance over extended times (at least 60 d) (**Figure** **15**a). A bare stent and an SeDPA‐PPAam‐coated stent were implanted bilaterally in iliac arteries of a rabbit for four weeks and significantly improved endothelial regeneration and anticoagulant property of SeDPA‐PPAam stent was shown (Figure 15b). Other organometallic compounds^126^ as well as metals (Cu^2+^, Fe^2+^, Co^2+^, Ni^2+^, Zn^2+^)^127^ can also decompose RSNOs and thus perform localized synthesis of NO. Zinc wires implanted into the bloodstream of a rat showed minimal cell deposition on the surface of the implant due to local generation of NO, while platinum wires (non‐NO‐releasing) demonstrated extensive cellular and fibrin coverage.^127^

Copper‐based MOFs are capable of generating NO from endogenous RSNO reservoirs. This property has led to the exploitation of copper‐based MOFs for the design of NO‐generating films, as reported recently by Neufeld et al.^128^, ^129^, ^130^ The films were fabricated by doping water‐soluble copper‐based MOFs to a range of materials (chitosan, poly(vinyl chloride), cotton fabric). Chitosan/MOF membranes were shown to generate nanomolar concentrations of NO and the MOF‐catalyzed generation of NO can be repeated up to four cycles. The authors also showed that MOF capability to release NO was similar for MOFs in powder form versus MOFs embedded in chitosan membranes. Interestingly, this is not the case for cotton/MOF surfaces, in which NO generation from cotton/MOF surfaces was slower (catalysis reached completion in 4 h for cotton/MOF vs 1.5 h for pristine MOFs). These findings showed that polymeric building blocks can be used to tune MOF‐mediated NO release profile.

Harding et al. assessed the in vitro and in vivo degradability of copper‐based MOFs, H_3_[(Cu_4_Cl)_3_‐(BTTri)_8_] (Cu‐BTTri), and showed that they maintained their crystalline structure after immersion in endothelial cell media for 72 h or after exposure to whole blood for 30 min.^131^ The minimal biodegradation of Cu‐BTTri means it has the potential to extend the lifetime of the materials to suit long‐term biomedical applications. The authors combined Cu‐BTTri with a biomedical grade PU to establish PU/Cu‐BTTri films. The catalytic activity of PU/Cu‐BTTri films was preserved even after a 30 min exposure to blood, generating NO from CysamNO solution with a flux rate of 0.75 × 10^−9^ m s^−1^ cm^−2^.

## 6 Conclusions and Future Perspectives

Recent progress in the delivery of NO is highly inspiring. In this review, we highlighted NO release parameters (NO payload, maximum NO flux, NO release half‐life, time required to reach maximum flux, and duration of NO release), however at times it is still challenging to compare the parameters between studies due to irregular reporting. Thorough characterizations must remain at the forefront of NO efficacy studies. Importantly, the actual NO levels achieved within a biological system or native tissues should be addressed to facilitate a more rapid clinical translation of NO‐based therapeutics. This leads to an aspect that needs academic attention, i.e., quantification of NO at the desired site in vivo. Fluorescent dyes may assist in visualization of NO and provide a simple absolute quantification of cellular NO concentration in vitro^132^ but to our knowledge, are not available as yet for in vivo quantification of NO. Chang et al. recently reported a peptide‐based NO sensor that can resolve nanomolar concentrations of NO while providing information within biological systems (detecting NO in response to physiological levels of shear stress).^133^ This sensor may be introduced intravenously to assess NO levels in the circulation or at specific tissues. This example demonstrates the type of sensors that stand to make impact for the development of personalized NO therapies.

Our presentation aimed to demonstrate diverse strategies designed to date to overcome the challenges associated with biological administration of NO. Some of these methods are early stage technologies, and some have progressed to clinical trials. Methods and technologies for optimized administration of NO are being developed by several companies. Most of these focus on optimization of administration of inhalable NO (including but not limited to the efforts of NitricGen, Inc. (recently acquired by AIT Therapeutics, Inc.), Bellerophon Therapeutics, Inc., Third Pole, Inc., Novoteris LLC, and INOmax). This technology revolves around a lightweight, portable, and economical device that is able to generate NO on demand, allowing for convenient home use for patients and eliminating the need for large high pressure NO cylinders in the hospital setting, a complex gas injection device, or a trained respiratory therapy staff. The development of inhalable NO aims to treat patients with respiratory conditions including pulmonary infections, pulmonary arterial hypertension, cystic fibrosis, or tuberculosis. Third Pole, Inc. technology was studied in lambs with acute pulmonary hypertension and the efficacy was compared with NO delivered from a cylinder (the clinical gold standard). This study showed that breathing electrically generated NO reversed pulmonary vasoconstriction at a level that is similar to NO from a tank (pulmonary arterial pressure reduced from 29 to 24 mmHg at 1 min after breathing NO, and to 21 mmHg after 4 min of NO inhalation).^134^ Novoteris LLC technology was assessed in eight cystic fibrosis patients (phase I clinical study). Patients inhaled 160 ppm of NO for two periods of 5 d, three times daily, and the study showed that breathing NO decreased microbial pathogen load in the airways of cystic fibrosis and increased lung function. This includes NO activity against antibiotic multiresistant microorganisms (3.5‐log reduction of *P. aeruginosa*, 12‐log reduction of *E. coli*, 1‐log reduction of methicillin resistant *S. aureus*, 4.5‐log reduction of *Mycobacterium abscessus*).^135^

Other NO delivery technologies developed by companies include: Novoclem who develops pulmonary administration of macromolecular prodrugs of NO, Origin who develops plasma‐based approach to generate NO for wound healing and treatment of soft tissue infections, and finally, Novan, Inc. is in clinical trials with macromolecular donors of NO in a hydrogel matrix for topical administration as anti‐inflammatory and/or antimicrobial agent.

At present, we are not aware of clinical trials or late‐stage preclinical development of NO‐releasing implantable biomaterials. To our eyes, academic developments presented above are highly successful. Translational prospects also appear good: many biomaterials discussed above were developed with knowledge of successful biomaterials for clinical use^136^, ^137^ and some NO donors are approved for use in humans. However, markets, e.g., cardiovascular stents or catheters (noted here as some of the examples where NO‐releasing biomaterials could find commercial utility) are occupied by successful products, and bringing an innovation to the market is hard.

From a different perspective, a trend worthy of specific attention is the shift of activity from localized NO delivery to site‐specific NO synthesis via enzyme‐prodrug therapy (using natural enzymes or nonproteinaceous enzyme mimics). This innovative methodology is unique and presents previously unavailable opportunities in personalized medicine. Specifically, precise, on‐demand control and up/downregulation of NO flux is achieved by the adjustment of the enzymatic output and the amount of externally administered NO prodrugs. Localized generation of NO via prodrug conversion has already been achieved using cardiovascular stents including sound in vivo evaluation. Translational prospects of EPT have been discussed recently in detail.^92^ One important aspect is that these technologies are advantageous in many ways, but to enter market would require approval of both the biocatalytic implant and the associated prodrug. In part, this consideration is addressed with the use of endogenous prodrugs, which are also advantageous in presenting essentially a life‐long supply of NO donor molecules.

Taken together, this review aims to illustrate that the materials and methods for delivery of NO have become highly powerful. However, there is significant room for improvement, specifically with regards to translational aspects of these methodologies. We hope that this review stimulates further research activity in this field.

## Conflict of Interest

The authors declare no conflict of interest.

## References

[1] D. E. Koshland , Science 1992, 258, 1861. 1470903 10.1126/science.1470903

[2] J. O. Lundberg , M. T. Gladwin , E. Weitzberg , Nat. Rev. Drug Discovery 2015, 14, 623. 26265312 10.1038/nrd4623

[3] C. Bogdan , Nat. Immunol. 2001, 2, 907. 11577346 10.1038/ni1001-907

[4] V. Calabrese , C. Mancuso , M. Calvani , E. Rizzarelli , D. A. Butterfield , A. M. G. Stella , Nat. Rev. Neurosci. 2007, 8, 766. 17882254 10.1038/nrn2214

[5] J. Y. H. Chang , W. D. Stamer , J. Bertrand , A. T. Read , C. M. Marando , C. R. Ethier , D. R. Overby , Am. J. Physiol. Cell Physiol. 2015, 309, C205. 26040898 10.1152/ajpcell.00347.2014 PMC4537932

[6] M. W. Radomski , R. M. J. Palmer , S. Moncada , Biochem. Biophys. Res. Commun. 1987, 148, 1482. 2825688 10.1016/s0006-291x(87)80299-1

[7] M. W. Vaughn , L. Kuo , J. C. Liao , Am. J. Physiol. 1998, 274, H2163. 9841542 10.1152/ajpheart.1998.274.6.H2163

[8] M. B. Witte , A. Barbul , Am. J. Surg. 2002, 183, 406. 11975928 10.1016/s0002-9610(02)00815-2

[9] J. Garthwaite , Eur. J. Neurosci. 2008, 27, 2783. 18588525 10.1111/j.1460-9568.2008.06285.x PMC2610389

[10] T. Akaike , H. Maeda , Immunology 2000, 101, 300. 11106932 10.1046/j.1365-2567.2000.00142.x PMC2327086

[11] G. F. Rimmelzwaan , M. Baars , P. de Lijster , R. A. M. Fouchier , A. Osterhaus , J. Virol. 1999, 73, 8880. 10482647 10.1128/jvi.73.10.8880-8883.1999 PMC112914

[12] R. N. Weinreb , B. S. Sforzolini , J. Vittitow , J. Liebmann , Ophthalmology 2016, 123, 965. 26875002 10.1016/j.ophtha.2016.01.019

[13] F. A. Medeiros , K. R. Martin , J. Peace , B. S. Sforzolini , J. L. Vittitow , R. N. Weinreb , Am. J. Ophthalmol. 2016, 168, 250. 27210275 10.1016/j.ajo.2016.05.012

[14] R. N. Weinreb , T. Ong , B. S. Sforzolini , J. L. Vittitow , K. Singh , P. L. Kaufman , V. S. Grp , Br. J. Ophthalmol. 2015, 99, 738. 25488946 10.1136/bjophthalmol-2014-305908 PMC4453588

[15] W. D. Stamer , Y. Lei , A. Boussommier‐Calleja , D. R. Overby , C. R. Ethier , Invest. Ophthalmol. Visual Sci. 2011, 52, 9438. 22039240 10.1167/iovs.11-7839 PMC3293415

[16] R. Hambrecht , K. Berra , K. J. Calfas , Circulation 2013, 127, e642. 23733971 10.1161/CIRCULATIONAHA.113.000821

[17] C. Opasich , G. Cioffi , A. Gualco , Curr. Heart Failure Rep. 2009, 6, 182. 10.1007/s11897-009-0026-4 19723460

[18] R. J. Barst , R. Channick , D. Ivy , B. Goldstein , Pulm. Circ. 2012, 2, 139. 22837854 10.4103/2045-8932.97589 PMC3401867

[19] M. B. Kaufman , Phys. Ther. 2018, 43, 22.

[20] P. N. Coneski , M. H. Schoenfisch , Chem. Soc. Rev. 2012, 41, 3753. 22362308 10.1039/c2cs15271a PMC3341472

[21] D. D. Thomas , L. A. Ridnour , J. S. Isenberg , W. Flores‐Santana , C. H. Switzer , S. Donzelli , P. Hussain , C. Vecoli , N. Paolocci , S. Ambs , C. A. Colton , C. C. Harris , D. D. Roberts , D. A. Wink , Free Radical Biol. Med. 2008, 45, 18. 18439435 10.1016/j.freeradbiomed.2008.03.020 PMC2572721

[22] D. D. Thomas , Redox Biol. 2015, 5, 225. 26056766 10.1016/j.redox.2015.05.002 PMC4473092

[23] J. S. Beckman , W. H. Koppenol , Am. J. Physiol. Cell Physiol. 1996, 271, C1424. 10.1152/ajpcell.1996.271.5.C1424 8944624

[24] D. D. Thomas , L. A. Ridnour , M. G. Espey , S. Donzelli , S. Ambs , S. P. Hussain , C. C. Harris , W. DeGraff , D. D. Roberts , J. B. Mitchell , D. A. Wink , J. Biol. Chem. 2006, 281, 25984. 16829532 10.1074/jbc.M602242200

[25] P. Pacher , J. S. Beckman , L. Liaudet , Physiol. Rev. 2007, 87, 315. 17237348 10.1152/physrev.00029.2006 PMC2248324

[26] U. Forstermann , N. Xia , H. G. Li , Circ. Res. 2017, 120, 713. 28209797 10.1161/CIRCRESAHA.116.309326

[27] N. Toda , K. Ayajiki , T. Okamura , Pharmacol. Rev. 2009, 61, 62. 19293146 10.1124/pr.108.000547

[28] L. Ying , L. J. Hofseth , Cancer Res. 2007, 67, 1407. 17308075 10.1158/0008-5472.CAN-06-2149

[29] D. Fukumura , S. Kashiwagi , R. K. Jain , Nat. Rev. Cancer 2006, 6, 521. 16794635 10.1038/nrc1910

[30] Y. Q. Wo , E. J. Brisbois , R. H. Bartlett , M. E. Meyerhoff , Biomater. Sci. 2016, 4, 1161. 27226170 10.1039/c6bm00271d PMC4955746

[31] N. Naghavi , A. de Mel , O. S. Alavijeh , B. G. Cousins , A. M. Seifalian , Small 2013, 9, 22. 23136136 10.1002/smll.201200458

[32] P. G. Wang , M. Xian , X. P. Tang , X. J. Wu , Z. Wen , T. W. Cai , A. J. Janczuk , Chem. Rev. 2002, 102, 1091. 11942788 10.1021/cr000040l

[33] M. R. Miller , I. L. Megson , Br. J. Pharmacol. 2007, 151, 305. 17401442 10.1038/sj.bjp.0707224 PMC2013979

[34] A. L. Fitzhugh , L. K. Keefer , Free Radical Biol. Med. 2000, 28, 1463. 10927170 10.1016/s0891-5849(00)00251-3

[35] F. Mazur , M. Bally , B. Städler , R. Chandrawati , Adv. Colloid Interface Sci. 2017, 249, 88. 28602208 10.1016/j.cis.2017.05.020

[36] B. S. Pattni , V. V. Chupin , V. P. Torchilin , Chem. Rev. 2015, 115, 10938. 26010257 10.1021/acs.chemrev.5b00046

[37] D. J. Suchyta , M. H. Schoenfisch , Mol. Pharmaceutics 2015, 12, 3569. 10.1021/acs.molpharmaceut.5b00248 26287799

[38] D. J. Suchyta , M. H. Schoenfisch , ACS Biomater. Sci. Eng. 2017, 3, 2136. 10.1021/acsbiomaterials.7b00255 PMC7164781 32309633

[39] K. Nakanishi , T. Koshiyama , S. Iba , M. Ohba , Dalton Trans. 2015, 44, 14200. 26200295 10.1039/c5dt02352a

[40] R. Chandrawati , B. Städler , A. Postma , L. A. Connal , S. F. Chong , A. N. Zelikin , F. Caruso , Biomaterials 2009, 30, 5988. 19683341 10.1016/j.biomaterials.2009.07.040

[41] P. Schattling , Y. Zhang , B. M. Teo , B. Städler , Rev. Cell Biol. Mol. Med. 2015, 1, 335.

[42] M. Talelli , M. Barz , C. J. F. Rijcken , F. Kiessling , W. E. Hennink , T. Lammers , Nano Today 2015, 10, 93. 25893004 10.1016/j.nantod.2015.01.005 PMC4398985

[43] Y. P. Li , K. Xiao , W. Zhu , W. B. Deng , K. S. Lam , Adv. Drug Delivery Rev. 2014, 66, 58. 10.1016/j.addr.2013.09.008 PMC3947689 24060922

[44] M. Gao , S. H. Liu , A. P. Fan , Z. Wang , Y. J. Zhao , RSC Adv. 2015, 5, 67041.

[45] Q. L. Song , S. W. Tan , X. T. Zhuang , Y. Y. Guo , Y. D. Zhao , T. T. Wu , Q. Ye , L. Q. Si , Z. P. Zhang , Mol. Pharmaceutics 2014, 11, 4118. 10.1021/mp5003009 25222114

[46] P. Kesharwani , A. K. Lyer , Drug Discovery Today 2015, 20, 536. 25555748 10.1016/j.drudis.2014.12.012 PMC4433832

[47] P. Kesharwani , K. Jain , N. K. Jain , Prog. Polym. Sci. 2014, 39, 268.

[48] S. Mignani , S. El Kazzouli , M. Bousmina , J. P. Majoral , Adv. Drug Delivery Rev. 2013, 65, 1316. 10.1016/j.addr.2013.01.001 23415951

[49] B. V. Worley , R. J. Soto , P. C. Kinsley , M. H. Schoenfisch , ACS Biomater. Sci. Eng. 2016, 2, 426. 10.1021/acsbiomaterials.6b00032 PMC7164776 32309632

[50] C. J. Backlund , B. V. Worley , M. H. Schoenfisch , Acta Biomater. 2016, 29, 198. 26478472 10.1016/j.actbio.2015.10.021 PMC4695967

[51] B. V. Worley , K. M. Schilly , M. H. Schoenfisch , Mol. Pharmaceutics 2015, 12, 1573. 10.1021/acs.molpharmaceut.5b00006 25873449

[52] B. V. Worley , D. L. Slomberg , M. H. Schoenfisch , Bioconjugate Chem. 2014, 25, 918. 10.1021/bc5000719 24797526

[53] Y. Lu , D. L. Slomberg , A. Shah , M. H. Schoenfisch , Biomacromolecules 2013, 14, 3589. 23962307 10.1021/bm400961r PMC3815563

[54] B. Sun , D. L. Slomberg , S. L. Chudasama , Y. Lu , M. H. Schoenfisch , Biomacromolecules 2012, 13, 3343. 23013537 10.1021/bm301109c PMC3482834

[55] P. N. Coneski , M. H. Schoenfisch , Org. Lett. 2009, 11, 5462. 19899748 10.1021/ol902282y PMC2788003

[56] Y. Piao , A. Burns , J. Kim , U. Wiesner , T. Hyeon , Adv. Funct. Mater. 2008, 18, 3745.

[57] L. Tang , J. J. Cheng , Nano Today 2013, 8, 290. 23997809 10.1016/j.nantod.2013.04.007 PMC3757135

[58] J. L. Vivero‐Escoto , I. I. Slowing , B. G. Trewyn , V. S.‐Y. Lin , Small 2010, 6, 1952. 20690133 10.1002/smll.200901789

[59] R. J. Soto , L. Yang , M. H. Schoenfisch , ACS Appl. Mater. Interfaces 2016, 8, 2220. 26717238 10.1021/acsami.5b10942 PMC4734612

[60] D. L. Slomberg , Y. Lu , A. D. Broadnax , R. A. Hunter , A. W. Carpenter , M. H. Schoenfisch , ACS Appl. Mater. Interfaces 2013, 5, 9322. 24006838 10.1021/am402618w

[61] Y. Lu , D. L. Slomberg , B. Sun , M. H. Schoenfisch , Small 2013, 9, 2189. 23362159 10.1002/smll.201201798

[62] A. W. Carpenter , D. L. Slomberg , K. S. Rao , M. H. Schoenfisch , ACS Nano 2011, 5, 7235. 21842899 10.1021/nn202054f PMC3225065

[63] A. W. Carpenter , B. V. Worley , D. L. Slomberg , M. H. Schoenfisch , Biomacromolecules 2012, 13, 3334. 22998760 10.1021/bm301108x PMC3482837

[64] D. Pissuwan , T. Niidome , M. B. Cortie , J. Controlled Release 2011, 149, 65. 10.1016/j.jconrel.2009.12.006 20004222

[65] P. Ghosh , G. Han , M. De , C. K. Kim , V. M. Rotello , Adv. Drug Delivery Rev. 2008, 60, 1307. 10.1016/j.addr.2008.03.016 18555555

[66] R. Chandrawati , M. M. Stevens , Chem. Commun. 2014, 50, 5431. 10.1039/c4cc00572d 24618788

[67] H. T. T. Duong , N. N. M. Adnan , N. Barraud , J. S. Basuki , S. K. Kutty , K. Jung , N. Kumar , T. P. Davis , C. Boyer , J. Mater. Chem. B 2014, 2, 5003. 10.1039/c4tb00632a 32261833

[68] L. A. Dykman , N. G. Khlebtsov , Chem. Sci. 2017, 8, 1719. 28451297 10.1039/c6sc03631g PMC5396510

[69] C. D. Walkey , J. B. Olsen , H. Guo , A. Emili , W. C. W. Chan , J. Am. Chem. Soc. 2012, 134, 2139. 22191645 10.1021/ja2084338

[70] J. Panyam , V. Labhasetwar , Adv. Drug Delivery Rev. 2012, 64, 61. 10.1016/s0169-409x(02)00228-4 12628320

[71] H. K. Makadia , S. J. Siegel , Polymers 2011, 3, 1377. 22577513 10.3390/polym3031377 PMC3347861

[72] G. Lautner , M. E. Meyerhoff , S. P. Schwendeman , J. Controlled Release 2016, 225, 133. 10.1016/j.jconrel.2015.12.056 PMC4769910 26763376

[73] H. Nurhasni , J. F. Cao , M. Choi , I. Kim , B. L. Lee , Y. J. Jung , J. W. Yoo , Int. J. Nanomedicine 2015, 10, 3065. 25960648 10.2147/IJN.S82199 PMC4411019

[74] T. K. Nguyen , R. Selvanayagam , K. K. K. Ho , R. X. Chen , S. K. Kutty , S. A. Rice , N. Kumar , N. Barraud , H. T. T. Duong , C. Boyer , Chem. Sci. 2016, 7, 1016. 28808526 10.1039/c5sc02769a PMC5531038

[75] Y. B. Huang , J. Liang , X. S. Wang , R. Cao , Chem. Soc. Rev. 2017, 46, 126. 27841411 10.1039/c6cs00250a

[76] Y. J. Cui , B. Li , H. J. He , W. Zhou , B. L. Chen , G. D. Qian , Acc. Chem. Res. 2016, 49, 483. 26878085 10.1021/acs.accounts.5b00530

[77] C. B. He , D. M. Liu , W. B. Lin , Chem. Rev. 2015, 115, 11079. 26312730 10.1021/acs.chemrev.5b00125

[78] A. Lowe , P. Chittajallu , Q. Gong , J. Li , K. J. Balkus , Microporous Mesoporous Mater. 2013, 181, 17.

[79] E. D. Bloch , W. L. Queen , S. Chavan , P. S. Wheatley , J. M. Zadrozny , R. Morris , C. M. Brown , C. Lamberti , S. Bordiga , J. R. Long , J. Am. Chem. Soc. 2015, 137, 3466. 25710124 10.1021/ja5132243

[80] S. Diring , D. O. Wang , C. Kim , M. Kondo , Y. Chen , S. Kitagawa , K. Kamei , S. Furukawa , Nat. Commun. 2013, 4, 2684. 24158008 10.1038/ncomms3684 PMC3826626

[81] A. N. Zelikin , ACS Nano 2010, 4, 2494. 20423067 10.1021/nn100634r

[82] J. Pant , J. Gao , M. J. Goudie , S. P. Hopkins , J. Locklin , H. Handa , Acta Biomater. 2017, 58, 421. 28579540 10.1016/j.actbio.2017.05.061 PMC5685542

[83] L. C. Xu , Y. Wo , M. E. Meyerhoff , C. A. Siedlecki , Acta Biomater. 2017, 51, 53. 28087484 10.1016/j.actbio.2017.01.030 PMC5346060

[84] A. M. W. Bartosch , R. Mathews , J. M. Tarbell , Biophys. J. 2017, 113, 101. 28700908 10.1016/j.bpj.2017.05.033 PMC5510764

[85] W. Yen , B. Cai , J. Yang , L. Zhang , M. Zeng , J. M. Tarbell , B. M. Fu , PLOS One 2015, 10, e0117133. 25575016 10.1371/journal.pone.0117133 PMC4289188

[86] R. Simon‐Walker , R. Romero , J. M. Staver , Y. Zang , M. M. Reynolds , K. C. Popat , M. J. Kipper , ACS Biomater. Sci. Eng. 2017, 3, 68. 10.1021/acsbiomaterials.6b00572 33429688

[87] Y. Q. Wo , E. J. Brisbois , J. F. Wu , Z. Li , T. C. Major , A. Mohammed , X. L. Wang , A. Colletta , J. L. Bull , A. J. Matzger , C. W. Xi , R. H. Bartlett , M. E. Meyerhoff , ACS Biomater. Sci. Eng. 2017, 3, 349. 28317023 10.1021/acsbiomaterials.6b00622 PMC5351555

[88] E. J. Brisbois , T. C. Major , M. J. Goudie , M. E. Meyerhoff , R. H. Bartlett , H. Handa , Acta Biomater. 2016, 44, 304. 27506125 10.1016/j.actbio.2016.08.009 PMC5045795

[89] E. J. Brisbois , M. Kim , X. W. Wang , A. Mohammed , T. C. Major , J. F. Wu , J. Brownstein , C. W. Xi , H. Handa , R. H. Bartlett , M. E. Meyerhoff , ACS Appl. Mater. Interfaces 2016, 8, 29270. 27734679 10.1021/acsami.6b08707 PMC5421361

[90] A. Colletta , J. F. Wu , Y. Q. Wo , M. Kappler , H. Chen , C. W. Xi , M. E. Meyerhoff , ACS Biomater. Sci. Eng. 2015, 1, 416. 26462294 10.1021/acsbiomaterials.5b00032 PMC4593359

[91] U. Förstermann , W. C. Sessa , Eur. Heart J. 2012, 33, 829. 21890489 10.1093/eurheartj/ehr304 PMC3345541

[92] B. Städler , A. N. Zelikin , Adv. Drug Delivery Rev. 2017, 118, 1. 10.1016/j.addr.2017.10.006 29054356

[93] B. Fejerskov , M. T. Jarlstad Olesen , A. N. Zelikin , Adv. Drug Delivery Rev. 2017, 118, 24. 10.1016/j.addr.2017.04.013 28457884

[94] A. C. Mendes , A. N. Zelikin , Adv. Funct. Mater. 2014, 24, 5202.

[95] R. Chandrawati , M. T. J. Olesen , T. C. C. Marini , G. Bisra , A. G. Guex , M. G. de Oliveira , A. N. Zelikin , M. M. Stevens , Adv. Healthcare Mater. 2017, 6, 1700385. 10.1002/adhm.201700385 PMC5590711 28699219

[96] A. Scomparin , H. F. Florindo , G. Tiram , E. L. Ferguson , R. Satchi‐Fainaro , Adv. Drug Delivery Rev. 2017, 118, 52. 10.1016/j.addr.2017.09.011 28916497

[97] S. K. Sharma , K. D. Bagshawe , Adv. Drug Delivery Rev. 2017, 118, 2. 10.1016/j.addr.2017.09.009 28916498

[98] P. Lehouritis , G. Hogan , M. Tangney , Adv. Drug Delivery Rev. 2017, 118, 8. 10.1016/j.addr.2017.09.012 28916496

[99] R. Mooney , A. Abdul Majid , J. Batalla , A. J. Annala , K. S. Aboody , Adv. Drug Delivery Rev. 2017, 118, 35. 10.1016/j.addr.2017.09.003 28916493

[100] F. Itel , P. S. Schattling , Y. Zhang , B. Städler , Adv. Drug Delivery Rev. 2017, 118, 94. 10.1016/j.addr.2017.09.006 28916495

[101] R. Walther , J. Rautio , A. N. Zelikin , Adv. Drug Delivery Rev. 2017, 118, 65. 10.1016/j.addr.2017.06.013 28676386

[102] J. E. Saavedra , T. R. Billiar , D. L. Williams , Y.‐M. Kim , S. C. Watkins , L. K. Keefer , J. Med. Chem. 1997, 40, 1947. 9207935 10.1021/jm9701031

[103] J. E. Saavedra , P. J. Shami , L. Y. Wang , K. M. Davies , M. N. Booth , M. L. Citro , L. K. Keefer , J. Med. Chem. 2000, 43, 261. 10649981 10.1021/jm9903850

[104] K. Sharma , A. Iyer , K. Sengupta , H. Chakrapani , Org. Lett. 2013, 15, 2636. 23659457 10.1021/ol400884v

[105] K. Sharma , K. Sengupta , H. Chakrapani , Bioorganic Med. Chem. Lett. 2013, 23, 5964. 10.1016/j.bmcl.2013.08.066 24050886

[106] X. J. Wu , X. P. Tang , M. Xian , P. G. Wang , Tetrahedron Lett. 2001, 42, 3779.

[107] T. B. Cai , D. Lu , M. Landerholm , P. G. Wang , Org. Lett. 2004, 6, 4203. 15524443 10.1021/ol048397p

[108] L. K. Keefer , ACS Chem. Biol. 2011, 6, 1147. 21932836 10.1021/cb200274r PMC3220281

[109] C. Chen , Y. Shi , S. Li , Q. Qi , L. Gu , J. Song , P. G. Wang , Arch. Pharm. 2006, 339, 366. 10.1002/ardp.200500262 16783837

[110] C. Chen , Y. Q. Shi , J. Song , Q. S. Qi , L. Gu , P. G. Wang , Biol. Pharm. Bull. 2006, 29, 1239. 16755024 10.1248/bpb.29.1239

[111] C. Chen , E. Zhang , Appl. Microbiol. Biotechnol. 2013, 97, 7377. 23801048 10.1007/s00253-013-5040-5

[112] L. Deng , E. Zhang , C. Chen , Arch. Pharmacal Res. 2013, 36, 619. 10.1007/s12272-013-0047-0 23494564

[113] C. A. Valdez , J. E. Saavedra , B. M. Showalter , K. M. Davies , T. C. Wilde , M. L. Citro , J. J. Barchi , J. R. Deschamps , D. Parrish , S. El‐Gayar , U. Schleicher , C. Bogdan , L. K. Keefer , J. Med. Chem. 2008, 51, 3961. 18533711 10.1021/jm8000482 PMC2574667

[114] Z. Wang , Y. Lu , K. Qin , Y. Wu , Y. Tian , J. Wang , J. Zhang , J. Hou , Y. Cui , K. Wang , J. Shen , Q. Xu , D. Kong , Q. Zhao , J. Controlled Release 2015, 210, 179. 10.1016/j.jconrel.2015.05.283 26004323

[115] A. de Mel , F. Murad , A. M. Seifalian , Chem. Rev. 2011, 111, 5742. 21663322 10.1021/cr200008n

[116] Y. Nie , K. Zhang , S. Zhang , D. Wang , Z. Han , Y. Che , D. Kong , Q. Zhao , Z. Han , Z. X. He , N. Liu , F. Ma , Z. Li , Acta Biomater. 2017, 63, 190. 28859902 10.1016/j.actbio.2017.08.037

[117] R. Chandrawati , J. Y. H. Chang , E. Reina‐Torres , C. Jumeaux , J. M. Sherwood , W. D. Stamer , A. N. Zelikin , D. R. Overby , M. M. Stevens , Adv. Mater. 2017, 29, 1604932. 10.1002/adma.201604932 PMC5400071 28221702

[118] B. Städler , A. D. Price , A. N. Zelikin , Adv. Funct. Mater. 2011, 21, 14.

[119] S.‐F. Chong , R. Chandrawati , B. Städler , J. Park , J. Cho , Y. Wang , Z. Jia , V. Bulmus , T. P. Davis , A. N. Zelikin , F. Caruso , Small 2009, 5, 2601. 19771568 10.1002/smll.200900906

[120] M. Kelm , BBA Bioenergetics 1999, 1411, 273. 10320663 10.1016/s0005-2728(99)00020-1

[121] J. S. Stamler , O. Jaraki , J. Osborne , D. I. Simon , J. Keaney , J. Vita , D. Singel , C. R. Valeri , J. Loscalzo , Proc. Natl. Acad. Sci. USA 1992, 89, 7674. 1502182 10.1073/pnas.89.16.7674 PMC49773

[122] W. Cha , M. E. Meyerhoff , Biomaterials 2007, 28, 19. 16959311 10.1016/j.biomaterials.2006.08.019

[123] Y. Weng , Q. Song , Y. Zhou , L. Zhang , J. Wang , J. Chen , Y. Leng , S. Li , N. Huang , Biomaterials 2011, 32, 1253. 21093045 10.1016/j.biomaterials.2010.10.039

[124] J. Yang , J. L. Welby , M. E. Meyerhoff , Langmuir 2008, 24, 10265. 18710268 10.1021/la801466e PMC2824255

[125] Z. Yang , Y. Yang , K. Xiong , X. Li , P. Qi , Q. Tu , F. Jing , Y. Weng , J. Wang , N. Huang , Biomaterials 2015, 63, 80. 26093790 10.1016/j.biomaterials.2015.06.016

[126] S. Hwang , M. E. Meyerhoff , J. Mater. Chem. 2008, 18, 1784.

[127] C. W. McCarthy , R. J. Guillory , J. Goldman , M. C. Frost , ACS Appl. Mater. Interfaces 2016, 8, 10128. 27031652 10.1021/acsami.6b00145

[128] M. J. Neufeld , A. Lutzke , J. B. Tapia , M. M. Reynolds , ACS Appl. Mater. Interfaces 2017, 9, 5139. 28164705 10.1021/acsami.6b14937 PMC6322424

[129] M. J. Neufeld , B. R. Ware , A. Lutzke , S. R. Khetani , M. M. Reynolds , ACS Appl. Mater. Interfaces 2016, 8, 19343. 27447022 10.1021/acsami.6b05948

[130] M. J. Neufeld , J. L. Harding , M. M. Reynolds , ACS Appl. Mater. Interfaces 2015, 7, 26742. 26595600 10.1021/acsami.5b08773

[131] J. L. Harding , J. M. Metz , M. M. Reynolds , Adv. Funct. Mater. 2014, 24, 7503.

[132] E. Eroglu , B. Gottschalk , S. Charoensin , S. Blass , H. Bischof , R. Rost , C. T. Madreiter‐Sokolowski , B. Pelzmann , E. Bernhart , W. Sattler , S. Hallström , T. Malinski , M. Waldeck‐Weiermair , W. F. Graier , R. Malli , Nat. Commun. 2016, 7, 10623. 26842907 10.1038/ncomms10623 PMC4743004

[133] J. Y. H. Chang , L. W. Chow , W. M. Dismuke , C. R. Ethier , M. M. Stevens , W. D. Stamer , D. R. Overby , Adv. Healthcare Mater. 2017, 6, 1700383. 10.1002/adhm.201700383 PMC5568941 28512791

[134] B. Yu , S. Muenster , A. H. Blaesi , D. B. Bloch , W. M. Zapol , Sci. Transl. Med. 2015, 7, 294ra107. 10.1126/scitranslmed.aaa3097 26136478

[135] C. Deppisch , G. Herrmann , U. Graepler‐Mainka , H. Wirtz , S. Heyder , C. Engel , M. Marschal , C. C. Miller , J. Riethmüller , Infection 2016, 44, 513. 26861246 10.1007/s15010-016-0879-x

[136] E. T. Pashuck , M. M. Stevens , Sci. Transl. Med. 2012, 4, 160sr4. 23152328 10.1126/scitranslmed.3002717

[137] E. S. Place , N. D. Evans , M. M. Stevens , Nat. Mater. 2009, 8, 457. 19458646 10.1038/nmat2441

